# Coronary heart disease and ischemic stroke polygenic risk scores and atherosclerotic cardiovascular disease in a diverse, population-based cohort study

**DOI:** 10.1371/journal.pone.0285259

**Published:** 2023-06-16

**Authors:** Allison Bebo, Jamie A. Jarmul, Mark J. Pletcher, Natalie R. Hasbani, David Couper, Vijay Nambi, Christie M. Ballantyne, Myriam Fornage, Alanna C. Morrison, Christy L. Avery, Paul S. de Vries

**Affiliations:** 1 Human Genetics Center, Department of Epidemiology, Human Genetics and Environmental Sciences, School of Public Health, The University of Texas Health Science Center at Houston, Houston, TX, United States of America; 2 Gillings School of Public Health, Department of Health Policy and Management, University of North Carolina, Chapel Hill, Chapel Hill, NC, United States of America; 3 School of Medicine, University of North Carolina, Chapel Hill, Chapel Hill, NC, United States of America; 4 Departments of Epidemiology and Biostatistics and Medicine, University of California, San Francisco, San Francisco, CA, United States of America; 5 Gillings School of Public Health, Department of Biostatistics, University of North Carolina, Chapel Hill, Chapel Hill, NC, United States of America; 6 Collaborative Studies Coordinating Center, University of North Carolina, Chapel Hill, Chapel Hill, NC, United States of America; 7 Baylor College of Medicine, Houston, TX, United States of America; 8 Michael E DeBakey Veterans Affairs Medical Center, Houston, TX, United States of America; 9 McGovern Medical School Institute of Molecular Medicine Research Center for Human Genetics, Houston, TX, United States of America; 10 Gillings School of Public Health, Department of Epidemiology, University of North Carolina, Chapel Hill, Chapel Hill, NC, United States of America; 11 Carolina Population Center, University of North Carolina–Chapel Hill, Chapel Hill, NC, United States of America; Brigham and Women’s Hospital and Harvard Medical School, UNITED STATES

## Abstract

The predictive ability of coronary heart disease (CHD) and ischemic stroke (IS) polygenic risk scores (PRS) have been evaluated individually, but whether they predict the combined outcome of atherosclerotic cardiovascular disease (ASCVD) remains insufficiently researched. It is also unclear whether associations of the CHD and IS PRS with ASCVD are independent of subclinical atherosclerosis measures. 7,286 White and 2,016 Black participants from the population-based Atherosclerosis Risk in Communities study who were free of cardiovascular disease and type 2 diabetes at baseline were included. We computed previously validated CHD and IS PRS consisting of 1,745,179 and 3,225,583 genetic variants, respectively. Cox proportional hazards models were used to test the association between each PRS and ASCVD, adjusting for traditional risk factors, ankle-brachial index, carotid intima media thickness, and carotid plaque. The hazard ratios (HR) for the CHD and IS PRS were significant with HR of 1.50 (95% CI: 1.36–1.66) and 1.31 (95% CI: 1.18–1.45) respectively for the risk of incident ASCVD per standard deviation increase in CHD and IS PRS among White participants after adjusting for traditional risk factors. The HR for the CHD PRS was not significant with an HR of 0.95 (95% CI: 0.79–1.13) for the risk of incident ASCVD in Black participants. The HR for the IS PRS was significant with an HR of 1.26 (95%CI: 1.05–1.51) for the risk of incident ASCVD in Black participants. The association of the CHD and IS PRS with ASCVD was not attenuated in White participants after adjustment for ankle-brachial index, carotid intima media thickness, and carotid plaque. The CHD and IS PRS do not cross-predict well, and predict better the outcome for which they were created than the composite ASCVD outcome. Thus, the use of the composite outcome of ASCVD may not be ideal for genetic risk prediction.

## Introduction

Both coronary heart disease (CHD) and ischemic stroke (IS) are caused by atherosclerosis, and therefore share many risk factors and intervention methods to lower their risk [1–3]. Risk assessment for atherosclerotic cardiovascular disease (ASCVD), a composite outcome including CHD and IS, is based on the 2013 Pooled Cohort Equation (PCE) and incorporates standard ASCVD risk factors like blood pressure, total cholesterol, and age. The PCE is widely implemented in clinical settings to facilitate early intervention with preventive therapies such as prescribing statins to lower low-density lipoprotein (LDL) cholesterol levels [4, 5].

To date, genome-wide association studies (GWAS) have identified 204 genome-wide significant genetic variants associated with CHD and 27 genetic variants associated with IS [6–10]. Polygenic risk scores (PRS) composed of the genetic variants discovered by GWAS can be used to estimate an individual’s burden of genetic risk and could be used to supplement existing risk prediction algorithms earlier in the primary prevention of ASCVD [11]. Recently, comprehensive polygenic risk scores (PRS) based on millions of genetic variants have been shown to be strong predictors of CHD and IS [11]. A recent study suggests that a CHD PRS is more predictive of CHD than any individual traditional risk factor defined by the PCE [12]. It is unclear the extent to which CHD and IS PRS predict the composite outcome of ASCVD as most previous studies have not included ASCVD in their analyses [11–19].

Furthermore, GWAS of CHD and IS have included predominantly participants of European ancestry [20, 21]. Several studies suggest that PRS derived from non-diverse GWAS do not generalize well across ancestries [22–24]. The generalizability of CHD and IS PRS based predominantly on genomic associations in White participants to Black participants has not yet been extensively explored, but the evidence to date suggests that there is a considerable attenuation of the association in African Americans [14, 25, 26]. Finally, most testing of PRS has focused on whether the PRS predict independent of traditional risk factors, so it is unknown to which extent the PRS also predict independent of measured markers of subclinical atherosclerosis such as ankle-brachial index (ABI), carotid intima media thickness (cIMT), and carotid plaque.

We examined whether two previously validated PRS for CHD and IS predict ASCVD outcomes in White and Black participants aged 45 to 64 years over a ten-year risk prediction period within the prospective Atherosclerosis Risk in Communities (ARIC) Study cohort [12, 13]. Additionally, this study investigated whether the CHD and IS PRS can cross-predict for IS and CHD outcomes, respectively, and whether these PRS are associated with incident ASCVD independent of measures of subclinical atherosclerosis, including ABI, cIMT, and carotid plaque.

## Materials and methods

### Study design and subjects

The ARIC study is a prospective, population-based cohort study conducted among mostly White and Black participants from four centers in the United States [27]. In brief, 15,792 participants aged 45 to 64 years were enrolled from 1987 to 1989. Of those participants, 12,153 participants have genotype data available and consented to the study. After the baseline visit, three, triennial follow-up visits were conducted. A fifth visit occurred between 2011 to 2013, a sixth visit occurred between 2016 to 2017, and a seventh visit occurred between 2018 and 2019. The study participants were contacted annually (semi-annually from 2012) by telephone in order to ascertain their health status. All included participants provided written informed consent. The ARIC study was approved by the University of Mississippi Medical Center IRB, Wake Forest University Health Sciences IRB, University of Minnesota IRB, and John Hopkins University IRB.

**Fig 1:**
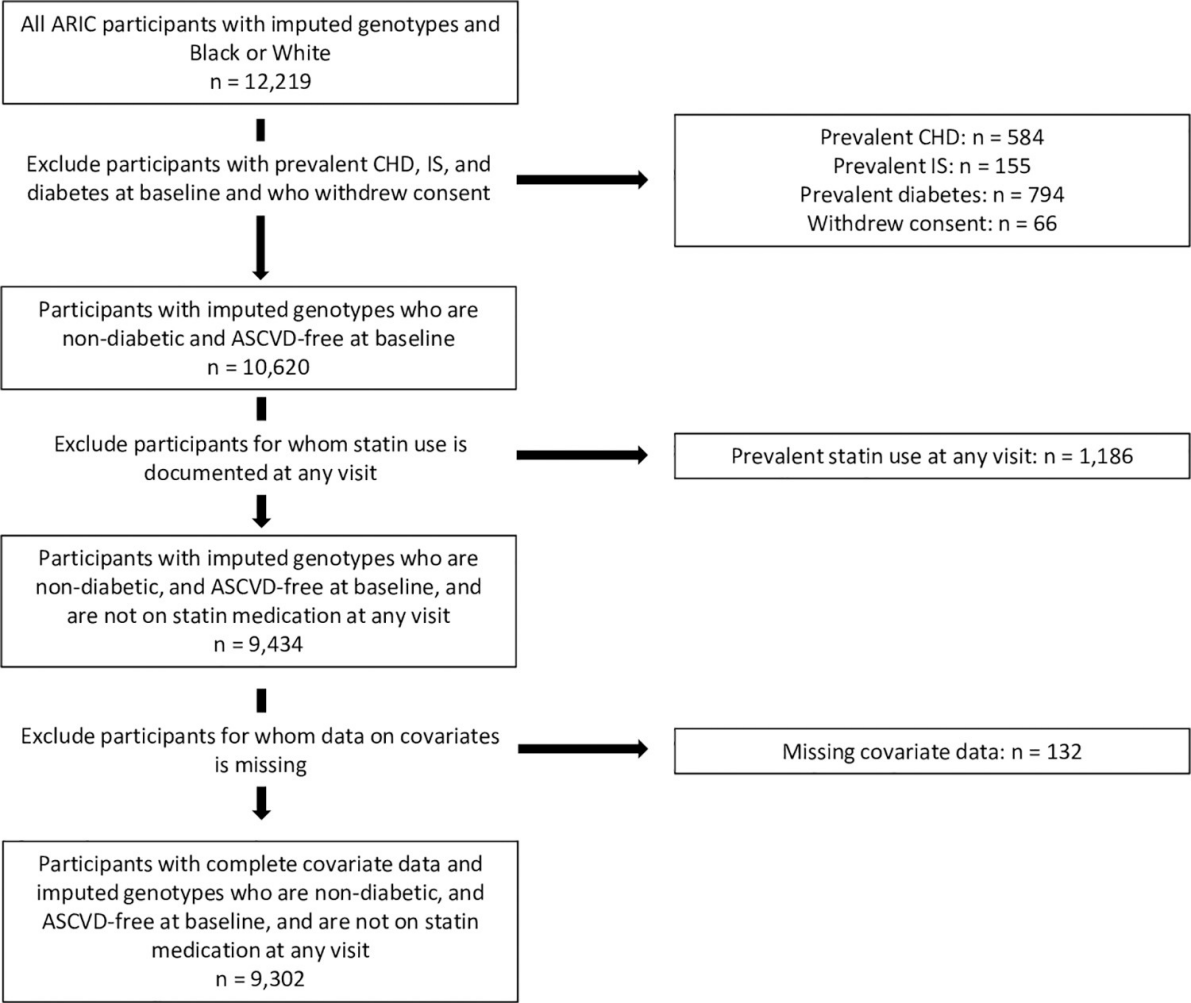
Flowchart of study population selection.

Participants were excluded if they experienced a CHD event or IS event prior to visit 1 (baseline). Prevalent CHD was defined at baseline in the ARIC study as MI diagnosed by an electrocardiogram test; any prior, self-reported cardiovascular-related surgeries; or a prior coronary angioplasty. Prevalent IS was defined as previous IS or transient ischemic attack [28, 29]. Additionally, all participants on statin therapy at visits one through four were excluded in order to examine the role of the PRS in the primary prevention of ASCVD, without the confounding influence of statin therapy on time-to-ASCVD. Participants with type 2 diabetes mellitus (T2DM) at the baseline exam and individuals with missing information for any covariates defined by the PCE were also excluded. T2DM was defined as a self-reported diagnosis of diabetes by a physician at baseline, medication use for T2DM, or fasting blood glucose ≥ 26 mg/dL or non-fasting blood glucose ≥ 200 mg/dL [30]. Covariates measured at the baseline exam included systolic blood pressure (SBP), total cholesterol, high density lipoprotein (HDL) cholesterol, smoking status, and anti-hypertensive medication use. The final study sample included 7,286 White participants and 2,016 Black participants (Fig 1).

### Atherosclerotic cardiovascular disease

Incident CHD and IS were determined through surveillance of local hospital discharge paperwork and death certificates, as well as phone interviews and surveys at follow-up visits [27]. ASCVD was defined as incident fatal and non-fatal CHD, revascularization (percutaneous coronary artery interventions including coronary artery bypass grafting and stent or balloon angioplasty), MI, and IS, and events were adjudicated by a committee of study physicians. The primary outcome measured in our study was time-to-ASCVD, defined as the time to the first CHD or IS event, whichever came first. The secondary outcomes measured in our study were time-to-CHD and time-to-IS. We restricted the study period from the baseline visit for each participant to ten years follow-up to match the PCE [27, 29, 31]. Patients were actively monitored for ASCVD events. Any events that were self-reported or discovered through surveillance were confirmed by ARIC physicians and investigators with medical records and insurance claims data [27]. Participants were assessed for first event, CHD or IS, in order to create the ASCVD outcome.

### Demographic and clinical risk factors

Demographics and medical history questionnaires were administered at baseline [27]. Race of the participants was self-reported [27]. Baseline total cholesterol, HDL cholesterol, SBP, smoking status, and anti-hypertensive medication use were treated as covariates in all statistical analyses. Blood pressure was collected three times with a random zero sphygmomanometer, and the average of the last two values was used [32]. Total and HDL cholesterol were measured using standard laboratory protocols [27]. Participants brought in all medications that they had taken in previous weeks at the baseline clinical exam and at all subsequent follow-up visits, and their types and doses were recorded with particular attention paid to statin and hypertension medication use [33].

### Subclinical measures of atherosclerosis

ABI, cIMT and carotid plaque were measured at baseline. ABI was measured using a Dinamap Model 1846 SX [34]. cIMT and carotid plaque were evaluated using carotid ultrasound by a trained professional. cIMT was measured within the distal common carotid artery, 1 cm proximal to the dilation of the carotid bulb. A total of 11 measurements were taken of the far wall in 1 mm increments, and the mean of the mean measurements of the left and right sides were estimated [35, 36]. During the ultrasound, the presence of carotid plaque was detected if two of the three following criteria were met: abnormal wall thickness in excess of 1.5 mm, abnormal shape, and abnormal wall texture [37]. Both ABI and cIMT were treated as continuous variables, and carotid plaque was treated as a dichotomous variable.

### Polygenic risk scores

ARIC participants were genotyped from plasma collected using the Affymetrix 6.0 array. After quality control, genotypes of 828,230 single nucleotide polymorphisms (SNPs) were available for 9,345 White participants and 2,874 Black participants. Genotype imputation was performed using sequencing data from the Trans-Omics for Precision Medicine (TOPMed) freeze 6 as a reference panel, covering roughly 230 million genetic variants for the Black participants, and roughly 239 million genetic variants for those White participants. Haplotype phasing and imputation was performed using the Michigan Imputation server, which is available at https://imputationserver.sph.umich.edu.

Weights from previously validated and independent metaGRSs for CHD and IS were used to calculate two, genome-wide polygenic risk scores in both White and Black ARIC participants [12, 13]. The weights and associated variant loci are available at https://www.pgscatalog.org/, with the IS PRS identification number PGS000039 and the CHD PRS identification number PGS000018. The CHD PRS is a metaGRS comprised of three PRS: 46,000 SNPs identified in a GWAS associated with cardiometabolic risk factors, a restricted PRS based on 202 genetic variants associated with CHD, and a genome-wide PRS from the same CHD GWAS [12]. The IS PRS is a more comprehensive metaGRS comprised of the three PRS previously mentioned that make up the CHD PRS as well as PRS associated with each of the stroke subtypes and eleven stroke risk factors [13].

The PRS were created in our population by weighting the dosage of the effect alleles dictated by these scores, by the beta coefficient associated with that SNP [38]. Of the 1,745,179 genetic variants that comprise the previously validated CHD PRS, there were 1,704,592 SNPs covered for White participants and 1,704,628 SNPs covered for Black participants within our dataset [12]. Of the 3,225,583 genetic variants that comprise the previously validated IS PRS, there were 3,156,481 SNPs covered for White participants and 3,156,554 SNPs covered for Black participants within our dataset [13]. The PRS were treated as continuous variables and analyzed per standard deviation (SD) increase in PRS, and stratified by race. Additionally, the PRS were categorized by quintiles of risk: the lower 20th percentile represented low risk, the 20th to the 80th percentile represented intermediate risk, and the upper 80th percentile represented high risk [12].

### Statistical analysis

The two sample t-test and the chi-square test were used to examine the differences in the two racial groups for each of the baseline characteristics included in the aforementioned PCE.

All analyses were stratified by race. The follow-up of participants who had not yet had an event was administratively censored at 10 years. Cox proportional hazard models were used to determine the association between each of the PRS and the time-to-ASCVD. All final models accounted for traditional risk factors, age, and gender. The first models evaluated the association of the PRS with just age and sex, and then with the aforementioned covariates defined by the PCE, and age and sex. Linear regression models tested the association of the PRS with ABI and cIMT, and a logistic regression tested the association of the PRS with carotid plaque. Three Cox models then evaluated the change in effect size of the PRS with the addition of each of the subclinical atherosclerosis measures one at a time, and a fourth included all three measures simultaneously. The PRS were analyzed as continuous predictors in all of the aforementioned models, and as categorical predictors in the primary analyses. The 4 primary association analyses included the association of the two PRS with ASCVD with TRFs in Black and White participants.

A likelihood ratio test was conducted in order to determine if the PRS contributed a statistically significant increase in the association of outcomes of ASCVD in each model. The improvement in discrimination with the addition of each of the PRS as well as both PRS simultaneously to all Cox proportional hazards models was assessed by Harrell’s c-statistic. The change in c-statistic when adding PRS was calculated by subtracting the c-statistic of each model without the PRS from the c-statistic of the corresponding model with the PRS. The net reclassification index (NRI) and the integrated discrimination index (IDI) were assessed between the traditional risk factors and the traditional risk factors plus each PRS as well as both PRS simultaneously for any significant results. The NRI and the IDI were also assessed for models with ABI, cIMT, carotid plaque, and the CHD and IS PRS to assess how well measures of subclinical atherosclerosis and the PRS together can predict ASCVD. The proportional hazards assumptions of the Cox proportional hazards models were assessed for all models using Schoenfeld residuals for continuous PRS and log-log plots for categorical PRS.

For sensitivity analyses, all the above analyses were repeated first using IS and then using CHD as the outcome instead of ASCVD. The Kaplan Meier method was used as a supplement to the Cox Proportional Hazards models in order to visually represent the survival of both White and Black participants by categorical PRS. We used a Bonferroni correction to adjust the significance threshold for multiple testing: a threshold of p<0.0125 was used to adjust for the 4 tests in our primary association analysis. All statistical analyses were performed in R version 3.6.2 and the survival package.

## Results

Table 1 displays the baseline characteristics of the participants, stratified by race. A total of 9,302 participants were included in the final study population, including 7,286 (77.7%) White participants and 2,016 (22.3%) Black participants. The mean age of White participants was 53.9 ± 5.7 years, and 52.8 ± 5.7 years for Black participants. The White participants were 54.9% female and Black participants were 62.1% female. During the first ten years of follow-up, White participants experienced 5.4 ASCVD events per 1,000 person-years, whereas Black participants experienced 6.8 ASCVD events per 1,000 person-years. For CHD and IS respectively, White participants experienced 4.3 and 4.3 events per 1,000 person-years, and Black participants experienced 1.3 and 2.5 events per 1,000 person-years. Table 2 shows the absolute incident ASCVD event rates, which also differed by race and PRS category. The spearman’s correlation between the two scores used in this study was 0.29 for White participants and 0.40 for Black participants.

**Table 1:** Summary statistics of baseline characteristics stratified by race, with means and standard deviations.

Variable of Interest	White Participants (n = 7,286)	Black Participants (n = 2,016)
**Genetic risk**		
Mean coronary heart disease polygenic risk score	353.3 ± 0.4	353.9 ± 0.3
Mean ischemic stroke polygenic risk score	188.0 ± 0.2	188.7 ± 0.2
**Cardiovascular disease risk factors at baseline**		
Gender (% female)	54.9%	62.1%
Age (years)	53.9 ± 5.7	52.8 ± 5.7
Total cholesterol (mmol/L)	5.42 ± 1.0	5.46 ± 1.1
High-density lipoprotein cholesterol (mmol/L)	1.35 ± 0.4	1.46 ± 0.5
Low-density lipoprotein cholesterol (mmol/L)	3.43 ± 0.9	3.47 ± 1.0
Systolic blood pressure (mmHg)	117.5 ± 16.5	127.4 ± 20.2
Diastolic blood pressure (mmHg)	71.5 ± 10.0	80.2 ± 12.1
Smokers (% current)	24.5%	30.3%
Anti-hypertensive medication (%)	20.3%	37.5%
Body mass index (kg/m2)	26.7 ± 4.7	29.2 ± 5.9
**Subclinical atherosclerosis measures**		
Ankle Brachial Index	1.14 ± 0.1	1.12 ± 0.1
Carotid intima media thickness	0.64 ± 0.1	0.68 ± 0.1
Carotid plaque (%)	32.7%	29.8%

**Table 2:** Number of atherosclerotic cardiovascular disease events and average follow-up time for 10-year follow-up, stratified by coronary heart disease and ischemic stroke polygenic risk score category.

**White Participants**				
**CHD PRS category**	**Number of ASCVD events**	**Average follow-up time for events (years) **	**Average follow-up time for non-events (years)**	**Average event rate per 1000 people per year**
Low risk (n = 1,458)	37	5.84 ± 3.09	9.84 ± 0.96	2.6
Intermediate risk (n = 4,371)	213	6.36 ± 2.59	9.79 ± 1.04	5.0
High risk (n = 1,457)	130	6.01 ± 2.87	9.82 ± 0.95	9.1
All (n = 7,286)	380	6.18 ± 2.74	9.80 ± 1.00	5.3
**IS PRS category**	**Number of ASCVD events**	**Average follow-up time for events (years)**	**Average follow-up time for non-events (years)**	**Average event rate per 1000 people per year**
Low risk (n = 1,458)	50	5.80 ± 2.86	9.85 ± 0.88	3.5
Intermediate risk (n = 4,371)	208	6.32 ± 2.73	9.80 ± 1.00	4.9
High risk (n = 1,457)	122	6.12 ± 2.70	9.76 ± 1.14	8.6
All (n = 7,286)	380	6.18 ± 2.74	9.80 ± 1.00	5.3
**Black Participants**				
**CHD PRS category**	**Number of ASCVD events**	**Average follow-up time for events (years) **	**Average follow-up time for non-events (years)**	**Average event rate per 1000 people per year**
Low risk (n = 404)	26	5.39 ± 2.62	9.84 ± 0.84	6.5
Intermediate risk (n = 1,209)	79	5.95 ± 2.59	9.79 ± 1.02	6.7
High risk (n = 403)	25	6.05 ± 2.52	9.75 ± 1.17	6.4
All (n = 2,016)	130	5.86 ± 2.57	9.79 ± 1.02	6.6
**IS PRS category**	**Number of ASCVD events**	**Average follow-up time for events (years)**	**Average follow-up time for non-events (years)**	**Average event rate per 1000 people per year**
Low risk (n = 404)	19	5.56 ± 2.42	9.78 ± 1.11	4.8
Intermediate risk (n = 1,209)	78	5.82 ± 2.61	9.80 ± 0.97	6.6
High risk (n = 403)	33	6.12 ± 2.60	9.78 ± 1.09	8.4
All (n = 2,016)	130	5.86 ± 2.57	9.79 ± 1.02	6.6

The hazard ratio (HR) for the CHD PRS was significant with an HR of 1.50 (95% CI: 1.36–1.66) for the risk of incident ASCVD per SD increase in PRS among White participants after adjusting for traditional risk factors (Table 3, S1 Fig). The same CHD PRS was not significantly associated with the time-to-ASCVD in Black participants with a hazard ratio of 0.95 (95% CI: 0.79–1.13) (S2 Fig). The HR for the IS PRS was significant with an HR of 1.31 (95% CI: 1.18–1.45) for the risk of ASCVD in White participants (S3 Fig). The HR for the IS PRS was significant with an HR of 1.26 (95% CI: 1.05–1.51) for the risk of incident ASCVD in Black participants (S4 Fig). Table 3 also shows the hazard ratios of the CHD and IS PRS associated with CHD and IS outcomes, respectively. The proportional hazards assumption was met for all models. S5 Fig shows the Schoenfeld residual plots for the primary models testing the association of the CHD and IS PRS with ASCVD, CHD, and IS.

**Table 3:** Association of CHD and IS PRS with time-to-ASCVD, time-to-CHD, and time-to-IS in the first 10 years of follow up given as hazard ratios (HR), in age and sex adjusted models with and without adjustment for the remaining traditional risk factors.

Atherosclerotic cardiovascular disease				
	**Adjusted for age and sex**		**Adjusted for traditional risk factors**	
	**HR (95% CI)**	**P-value**	**HR (95% CI)**	**P-value**
*White Participants (Ncases = 380*, *N = 7286)*				
CHD PRS	1.58 (1.43, 1.74)	**2.00E-16**	1.50 (1.36, 1.66)	**5.08E-15**
IS PRS	1.43 (1.30, 1.58)	**5.57E-13**	1.31 (1.18, 1.45)	**2.15E-07**
*Black Participants (Ncases = 130*, *N = 2016)*				
CHD PRS	1.01 (0.85, 1.19)	0.952	0.95 (0.79, 1.13)	0.55
IS PRS	1.36 (1.13, 1.63)	**9.65E-04**	1.26 (1.05, 1.51)	**0.012**
**Coronary heart disease**				
	**Adjusted for age and sex**		**Adjusted for traditional risk factors**	
	**HR (95% CI)**	**P-value**	**HR (95% CI)**	**P-value**
*White Participants (Ncases = 309*, *N = 7286)*				
CHD PRS	1.63 (1.46, 1.83)	**2.00E-16**	1.56 (1.39, 1.74)	**1.85E-14**
IS PRS	1.17 (1.05, 1.30)	**0.00507**	1.05 (0.94, 1.18)	0.39
*Black Participants (Ncases = 90*, *N = 2016)*				
CHD PRS	1.11 (0.90, 1.37)	0.322	1.05 (0.85, 1.30)	0.63
IS PRS	1.37 (1.10, 1.71)	**0.00486**	1.27 (1.02, 1.58)	**0.031**
**Ischemic stroke**				
	**Adjusted for age and sex**		**Adjusted for traditional risk factors**	
	**HR (95% CI)**	**P-value**	**HR (95% CI)**	**P-value**
*White participants (Ncases = 85*, *N = 7286)*				
CHD PRS	1.41 (1.14, 1.74)	**0.00159**	1.33 (1.08, 1.65)	**0.0082**
IS PRS	3.01 (2.47, 3.68)	**2.00E-16**	2.88 (2.34, 3.54)	**2.00E-16**
*Black participants (Ncases = 50*, *N = 2016)*				
CHD PRS	0.83 (0.63, 1.09)	0.179	0.79 (0.60, 1.05)	0.11
IS PRS	1.51 (1.12, 2.04)	**0.00648**	1.42 (1.06, 1.91)	**0.019**

The HR for incident ASCVD by the high and low categorical CHD and IS PRS groups are in S1 Table. The intermediate risk level was the reference level for all models. After adjusting for traditional risk factors, the low-risk CHD category was associated with an HR of 0.52 (95% CI: 0.36, 0.73) for risk of incident ASCVD, and the high-risk category was associated with an HR of 1.78 (95%: 1.43, 2.22) for risk of incident outcomes of ASCVD in White participants. The high risk IS PRS category alone was significant with an HR of 1.54 (95% CI: 1.23–1.93) for risk of incident ASCVD in White participants as well. No categories of the two PRS were significantly associated with incident ASCVD in Black participants. S1 Table also includes the HR by risk category for incident CHD and IS. The proportional hazards assumption was met for all models. S6 Fig shows the log-log plots for the categorical models testing the association of the CHD and IS PRS with ASCVD, CHD, and IS.

Table 4 shows the NRI, IDI, and Harrell’s c-statistic changes for each of our primary models. For White participants, the NRI estimate for the addition of the CHD PRS was 0.111 (95% CI: 0.042–0.185) and the IS PRS was 0.042 (95% CI: -0.020–0.101). The IDI estimate for the addition of the CHD PRS was 0.016 (95% CI: 0.008–0.025) and the IS PRS was 0.007 (95% CI: 0.003–0.015). Harrell’s c-statistic increased from 0.766 to 0.784 with the addition of the CHD PRS, and to 0.773 with the addition of the IS PRS. For Black participants, the NRI estimate for the addition of the CHD PRS was 0.016 (95% CI: -0.047–0.080) and the IS PRS was 0.020 (95% CI: -0.051–0.128). The IDI estimate for the addition of the CHD PRS was 0.001 (95% CI: -0.001–0.011) and the IS PRS was 0.004 (95% CI: -0.001–0.015). Harrell’s c-statistic did not change with the addition of the CHD PRS, and increased from 0.792 to 0.799 with the addition of the IS PRS.

**Table 4:** Reclassification, given by the net reclassification index (NRI) and integrated discrimination index (IDI), and changes in discrimination upon the addition of the CHD and IS PRS to models with traditional risk factors in White and Black participants.

**White participants**				
	**Time to first ASCVD event ~ established risk factors + CHD PRS**			
**NRI**	**Estimate**	**95% CI**	**IDI (95% CI)**	**c-statistic difference**
NRI	0.111	(0.042, 0.185)	0.016 (0.008, 0.025)	0.018
NRI +	0.092	(0.030, 0.163)		
NRI -	0.019	(0.007, 0.034)		
	**Time to first ASCVD event ~ established risk factors + IS PRS**			
**NRI**	**Estimate**	**95% CI**	**IDI (95% CI)**	**c-statistic difference**
NRI	0.042	(-0.020, 0.101)	0.007 (0.003, 0.015)	0.007
NRI +	0.028	(-0.026, 0.086)		
NRI -	0.014	(-0.001, 0.026)		
**Black participants**				
	**Time to first ASCVD event ~ established risk factors + CHD PRS**			
**NRI**	**Estimate**	**95% CI**	**IDI (95% CI)**	**c-statistic difference**
NRI	0.016	(-0.047, 0.080)	0.001 (-0.001, 0.011)	-1.00E-04
NRI +	0.015	(-0.050, 0.075)		
NRI -	0.001	(-0.011, 0.018)		
	**Time to first ASCVD event ~ established risk factors + IS PRS**			
**NRI**	**Estimate**	**95% CI**	**IDI (95% CI)**	**c-statistic difference**
NRI	0.020	(-0.051, 0.128)	0.004 (-0.001, 0.015)	0.007
NRI +	0.015	(-0.055, 0.119)		
NRI -	0.005	(-0.013, 0.028)		

When we examined the effects of the PRS in the context of markers of sub-clinical atherosclerosis in White participants, the HR for the CHD PRS was significant with an HR of 1.53 (95% CI: 1.36–1.73) and the HR for the IS PRS was significant with an HR of 1.37 (95% CI: 1.21–1.54) for risk of incident ASCVD, after adjusting for traditional risk factors as well as ABI, cIMT, and carotid plaque (S2 Table). cIMT and carotid plaque were significantly associated with a 2.70-fold and 1.75-fold increase in outcomes of ASCVD (95% CI: 1.17–6.24; 1.35–2.25) after adjusting for traditional risk factors, and the CHD and IS PRS (S3 Table).

S3 Table also includes models showing the association of ABI, cIMT, and carotid plaque with ASCVD, as well as the discrimination of models with ABI, cIMT, carotid plaque, and the CHD and IS PRS added separately and then together compared to a model with TRFs alone. The NRI estimate for the addition of ABI, cIMT, and carotid plaque was 0.100 (95% CI: 0.008–0.183), with an IDI estimate of 0.013 (95% CI: 0.008–0.026) and a c-statistic change of 0.016. The NRI estimate for the addition of the CHD PRS with ABI, cIMT, and carotid plaque was 0.176 (95% CI: 0.085–0.272), with an IDI estimate of 0.031 (95% CI: 0.021–0.051) and a c-statistic change of 0.034. The NRI estimate for the addition of the IS PRS and ABI, cIMT, and carotid plaque was 0.117 (95% CI: 0.036–0.216), with an IDI estimate of 0.024 (95% CI: 0.014–0.044) and a c-statistic change of .022.

## Discussion

In our study, we found that the CHD PRS and IS PRS predicted ASCVD independent of traditional risk factors in White participants, but only the IS PRS predicted ASCVD in Black participants. Additionally, we found that the CHD and IS PRS predicted incident ASCVD independent of measures of subclinical atherosclerosis in White participants. Finally, we found that neither the CHD nor the IS PRS cross-predicts well: that is, the CHD PRS did not predict IS as well as it predicted CHD, and vice versa.

African Americans have a higher incidence of IS than other ASCVD outcomes, as well as proven race-based health disparities in the United States [39, 40]. Inclusion of IS as an outcome in the combined outcome of ASCVD therefore aids in determining if the PRS generalize fully to the medical needs of diverse populations. Unfortunately, the performance of the CHD PRS in Black participants was poor. In contrast, the performance of the IS PRS in Black participants was more comparable to its performance in White participants. This is likely because the MEGASTROKE consortium has made considerable strides in including transethnic populations in their GWAS compared to the CARDIoGRAMplusC4D consortium [12, 13, 41, 42]. Additionally, it could be due in part to the inclusion of the GWAS of many risk factors in the IS PRS that may make the PRS more transferable to other ethnic groups. The PRS are not fully generalizable to other populations outside of European ethnic groups because of differing allele frequencies, linkage disequilibrium patterns, and potentially different causal variants [14, 23, 43, 44]. Performance of PRS derived from GWAS in mixed or European ancestry is usually poorest in African ancestry populations, because these populations have been particularly underrepresented in GWAS and also have the most diverging linkage disequilibrium patterns [45, 46]. The poor prediction in African Americans could exacerbate current race-based health disparities as their risk is not fully captured by genetic risk prediction [45]. However, ongoing GWAS of CAD will increase the number of included Black and Hispanic participants [47]. Future studies should continue this trend towards greater diversity to prevent PRS from widening existing health disparities.

The composite outcome of ASCVD is used in the primary prevention setting because both CHD and IS are caused by atherosclerosis and share a set of modifiable risk factors, including lipid levels [1]. This shared etiology may not fully extend to genetic risk: Malik et al. reported a moderate genetic correlation between CHD and IS of 0.51, and in our study the correlation between the two PRS was lower at r<0.4 [10]. The association of the PRS with incident ASCVD outcomes stems primarily from an association with their respective outcome, but also from a weaker association with the other components of ASCVD, reflecting this shared genetic background of CHD and stroke through atherosclerosis [48–52]. However, each PRS better predicts the outcome for which they were created than the broad outcome of ASCVD, which is consistent with the findings by Elliot, et al [18]. These results suggest that CHD and IS individually may be more clinically relevant outcomes for genetic risk prediction than the composite outcome of ASCVD. Conversely, the PRS could be strengthened by performing GWAS on the composite outcome of ASCVD instead of its component outcomes individually.

The CHD and IS PRS predicted incident ASCVD independent of the three measures of subclinical atherosclerosis (ABI, cIMT, and carotid plaque). Furthermore, the addition of the three measures of subclinical atherosclerosis in a model with the CHD and IS PRS to predict incident ASCVD did modestly improve discrimination compared to the PRS alone and the subclinical atherosclerosis measures alone. This suggests that PRS can be complementary to measures of subclinical atherosclerosis in the clinical setting for White patients. However, coronary artery calcification (CAC) measurements were not included in the subclinical atherosclerosis measurements at baseline in the ARIC study, although CAC scores better predict CHD than other measures of subclinical atherosclerosis [53]. A follow-up study in a cohort with CAC measurements at baseline should be considered in addition to this study, since CAC is currently the only measure of subclinical atherosclerosis that is implemented in risk prediction guidelines [54].

The major strengths of this study are that it was conducted using a well-established, prospective, and diverse cohort study in which ASCVD outcomes were carefully documented. Additionally, previous studies have shown that the PRS used in this study were state of the art and perform better than other, less comprehensive types of genetic risk scores [12, 14, 17]. Finally, this was one of the first studies to use the composite outcome of ASCVD as the primary outcome [18, 19].

The limitations are the underrepresentation of Black participants in GWAS, and the limited data available in Black participants within the ARIC study. Although Black participants were included in the study, the sample size was relatively low, and this may have affected the power of analyses restricted to Black participants. Genotyping arrays, including the Affymetrix 6.0 used here, were primarily constructed for populations of European ancestry. In populations like African Americans that are proportionally underrepresented in the discovery of common marker SNPs and have different linkage disequilibrium patterns, genotyping arrays may be underpowered to cover all common variants [55–57]. Both of the PRS used for this study were validated in participants of European ancestry from the UK Biobank, so their generalizability to different ethnicities may be limited [12, 13]. Finally, the ARIC study contributed data to the MEGASTROKE and CARDIoGRAMplusC4D consortiums, which could bias our results.

This study found that CHD and IS PRS predict ASCVD independent of traditional risk factors and measures of subclinical atherosclerosis in White ARIC participants, with reduced prediction in Black ARIC participants. Future discovery GWAS should include more diverse participants in order to ensure that PRS can be applied to primary prevention in an equitable manner. Additionally, this study suggests that CHD and IS individually may be more clinically relevant outcomes for genetic risk prediction in the clinical setting than the composite outcome of ASCVD.

## Supporting information

S1 Fig Kaplan-Meier survival curves for time-to-ASCVD in White participants, stratified by CHD PRS category. (PDF)

S2 Fig Kaplan-Meier survival curves for time-to-ASCVD in Black participants, stratified by CHD PRS category. (PDF)

S3 Fig Kaplan-Meier survival curves for time-to-ASCVD in White participants, stratified by IS PRS category. (PDF)

S4 Fig Kaplan-Meier survival curves for time-to-ASCVD in Black participants, stratified by IS PRS category. (PDF)

S5 Fig Schoenfeld residuals for the Cox proportional hazards models testing the association of the CHD and IS PRS with ASCVD, CHD, and IS. (PDF)

S6 Fig Log-log plots for the Cox proportional hazards models testing the association of the CHD and IS PRS with ASCVD, CHD, and IS. (PDF)

S1 Table Association of categorical PRS with time-to-ASCVD, time-to-CHD, and time-to-IS in the first 10 years of follow-up. (PDF)

S2 Table Association of CHD and IS PRS with incident ASCVD, CHD, and IS in the first 10 years of follow-up, after adjusting for traditional risk factors, ABI, cIMT, and carotid plaque. (PDF)

S3 Table Association of ABI, cIMT, and carotid plaque with incident ASCVD in the first 10 years of follow-up, adjusting for traditional risk factors and the CHD and IS PRS. Reclassification, given by the net reclassification index (NRI) and integrated discrimination index (IDI), and changes in discrimination upon the addition of the CHD PRS, IS PRS, ABI, cIMT, and carotid plaque to models with traditional risk factors in White participants.
(PDF)
