# Supplementary material for: Coronary heart disease and ischemic stroke polygenic risk scores and atherosclerotic cardiovascular disease in a diverse, population-based cohort study
Source: PLoS One. 2023 Jun 16;18(6):e0285259. doi: 10.1371/journal.pone.0285259 (PMC10275447; doi:10.1371/journal.pone.0285259)

S3 Fig. Kaplan-Meier survival curves for time-to-ASCVD in White participants, stratified by IS PRS category.

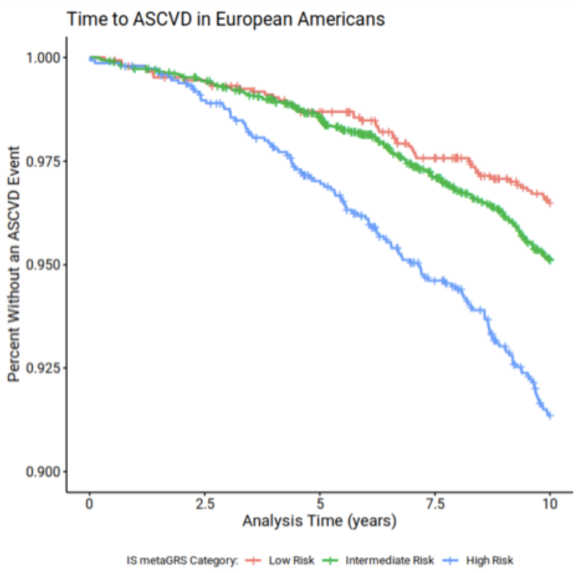

Supplement: S3 Fig — (PDF) [file pone.0285259.s003.pdf]
