# Supplementary material for: Coronary heart disease and ischemic stroke polygenic risk scores and atherosclerotic cardiovascular disease in a diverse, population-based cohort study
Source: PLoS One. 2023 Jun 16;18(6):e0285259. doi: 10.1371/journal.pone.0285259 (PMC10275447; doi:10.1371/journal.pone.0285259)

S5 Fig. Schoenfeld residuals for the Cox proportional hazards models testing the association of the CHD and IS PRS with ASCVD, CHD, and IS.

A: Schoenfeld residuals for the Cox proportional hazards model testing the association of the CHD PRS with ASCVD in White participants, adjusting for age and gender.

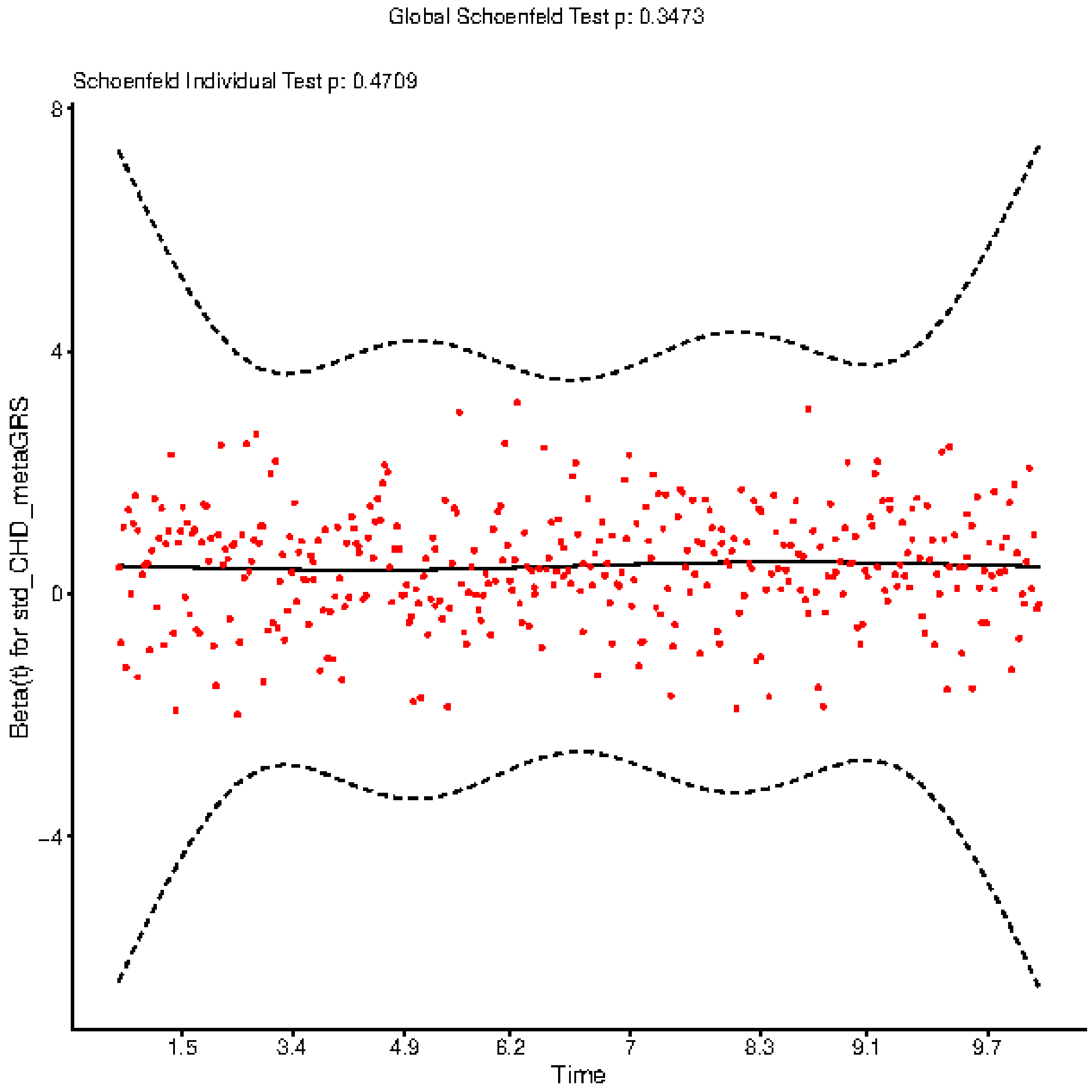

B: Schoenfeld residuals for the Cox proportional hazards model testing the association of the CHD PRS with ASCVD in White participants, adjusting for age, gender, and traditional risk factors.

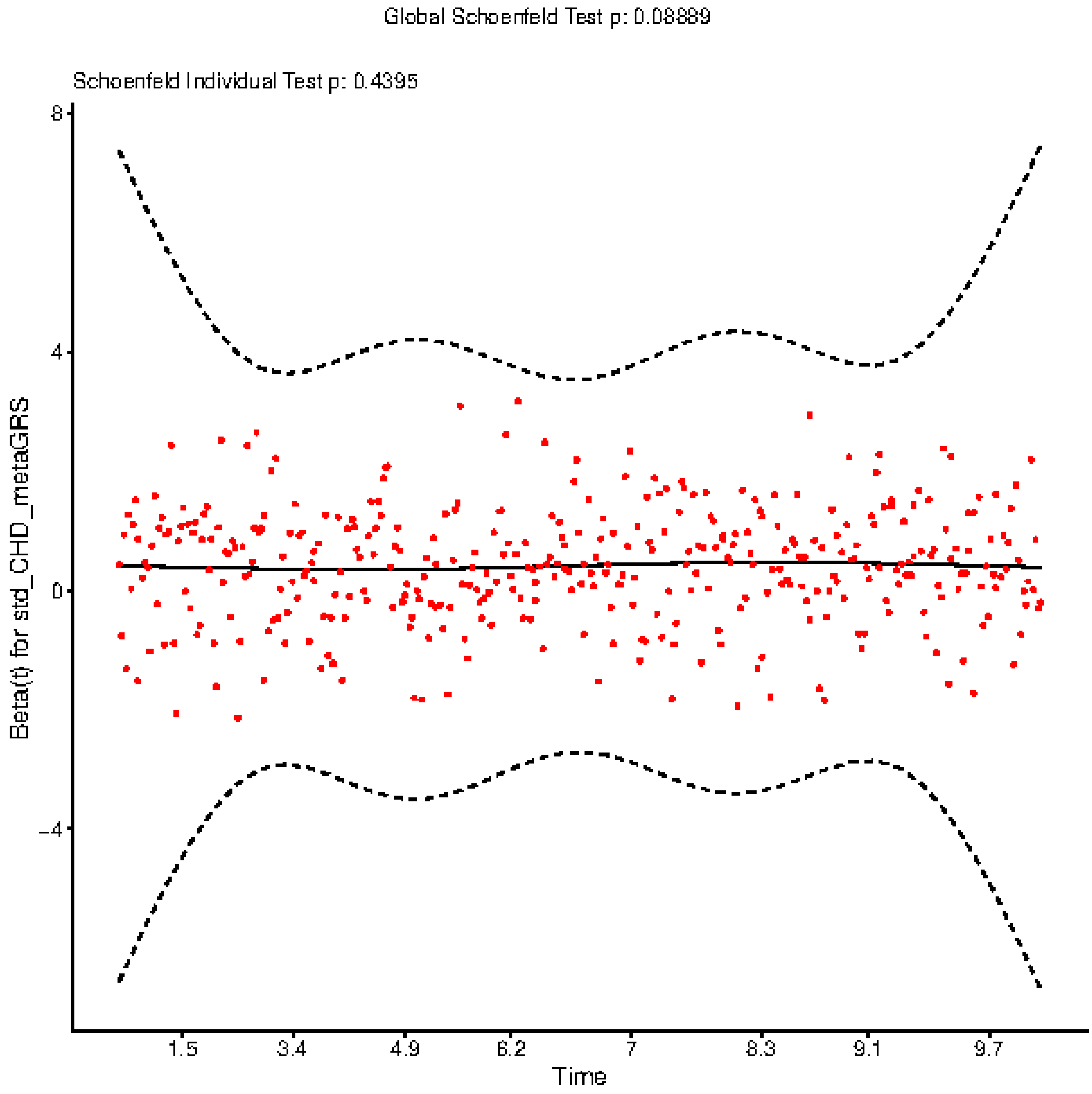

C: Schoenfeld residuals for the Cox proportional hazards model testing the association of the IS PRS with ASCVD in White participants, adjusting for age and gender.

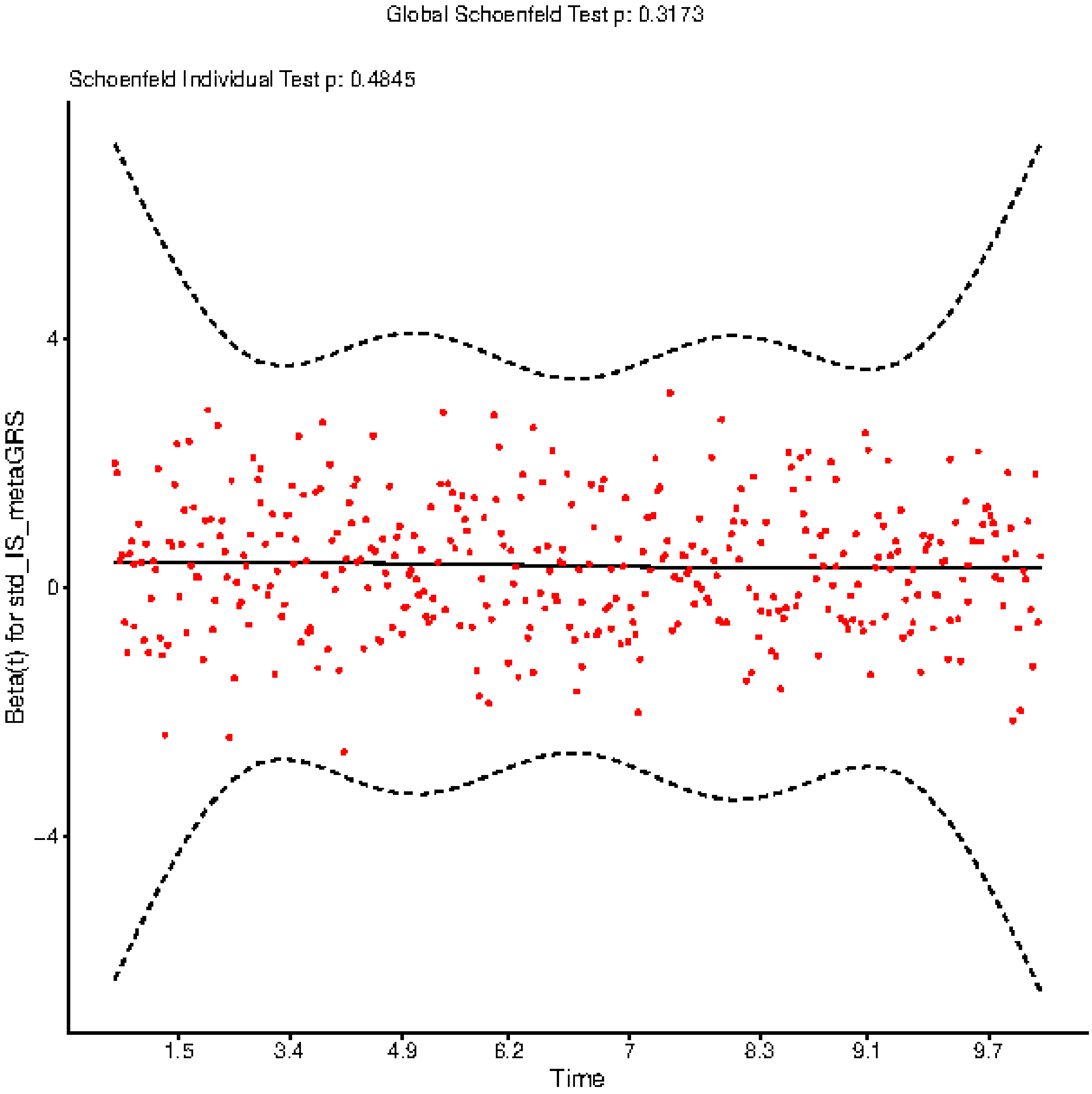

D: Schoenfeld residuals for the Cox proportional hazards model testing the association of the IS PRS with ASCVD in White participants, adjusting for age, gender, and traditional risk factors.

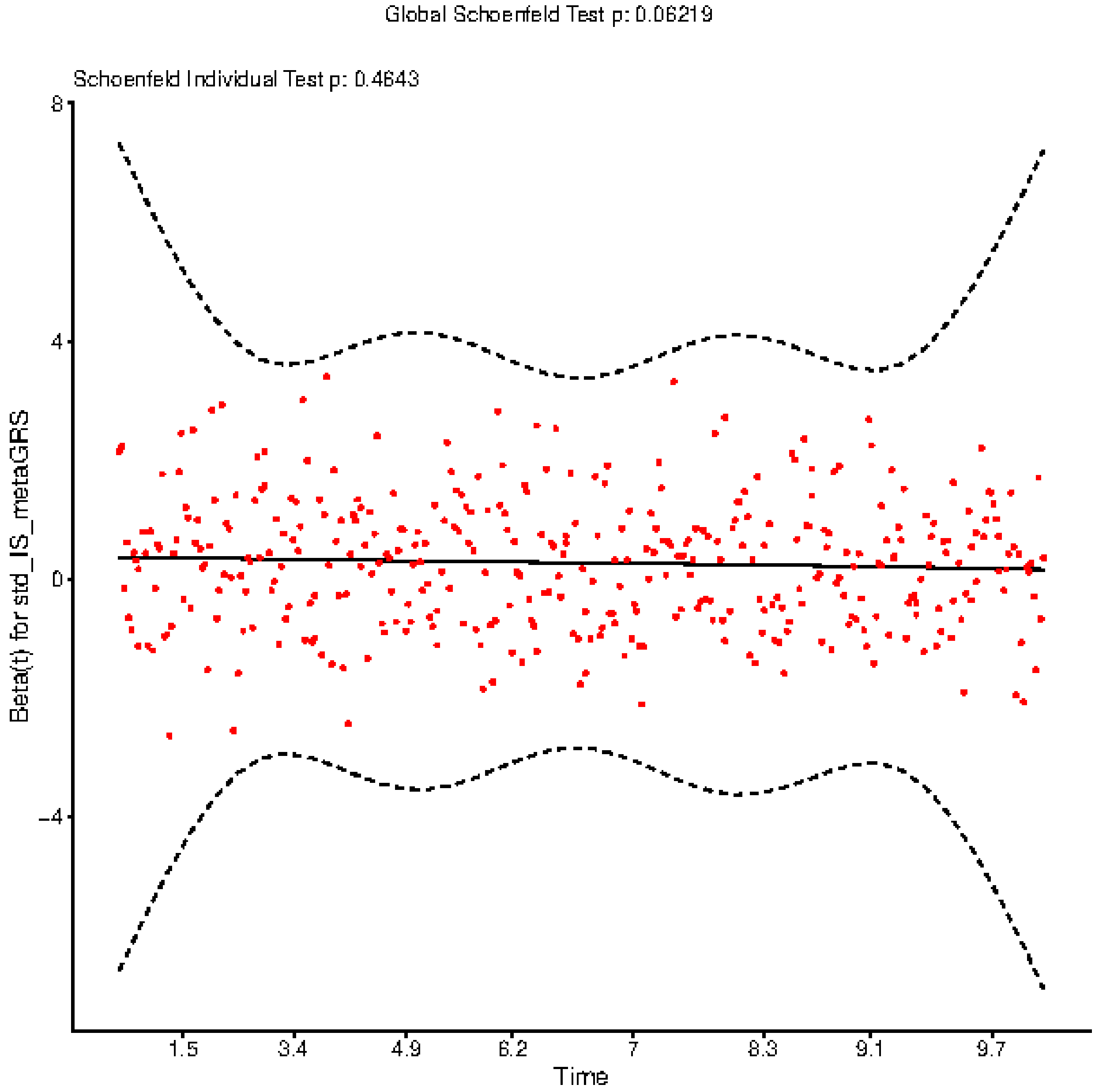

E: Schoenfeld residuals for the Cox proportional hazards model testing the association of the CHD PRS with ASCVD in Black participants, adjusting for age and gender.

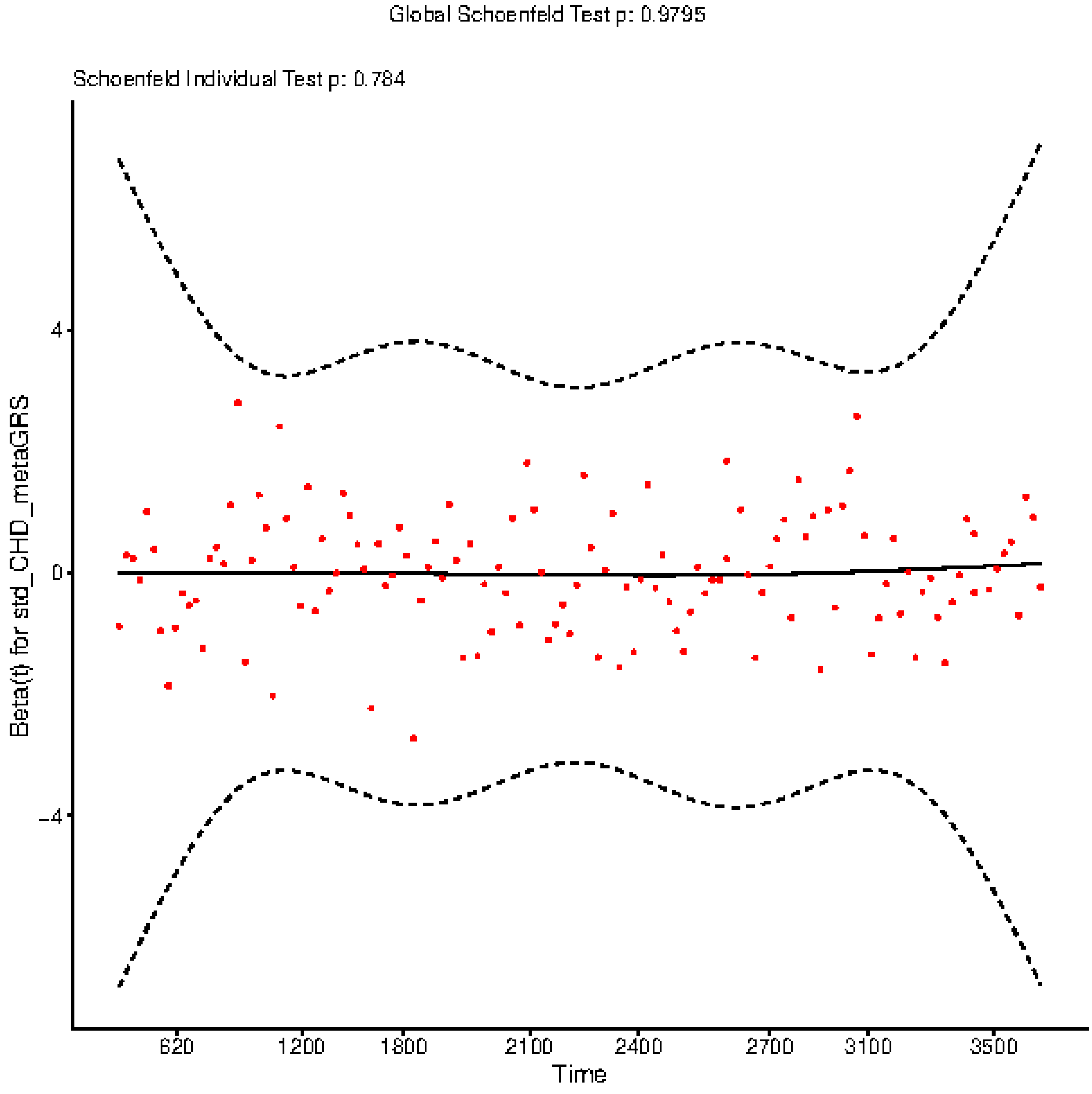

F: Schoenfeld residuals for the Cox proportional hazards model testing the association of the CHD PRS with ASCVD in Black participants, adjusting for age, gender, and traditional risk factors.

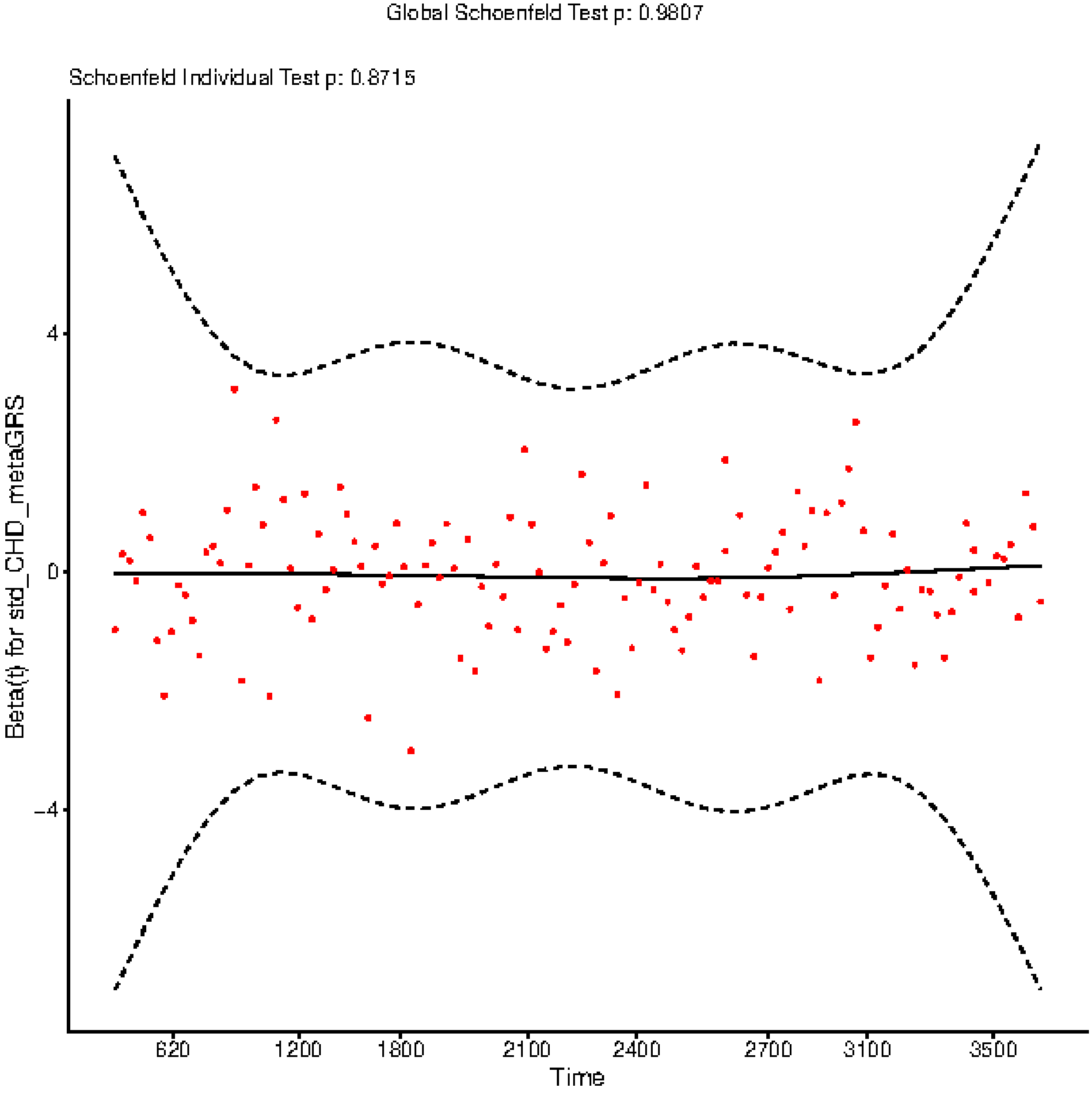

G: Schoenfeld residuals for the Cox proportional hazards model testing the association of the IS PRS with ASCVD in Black participants, adjusting for age and gender.

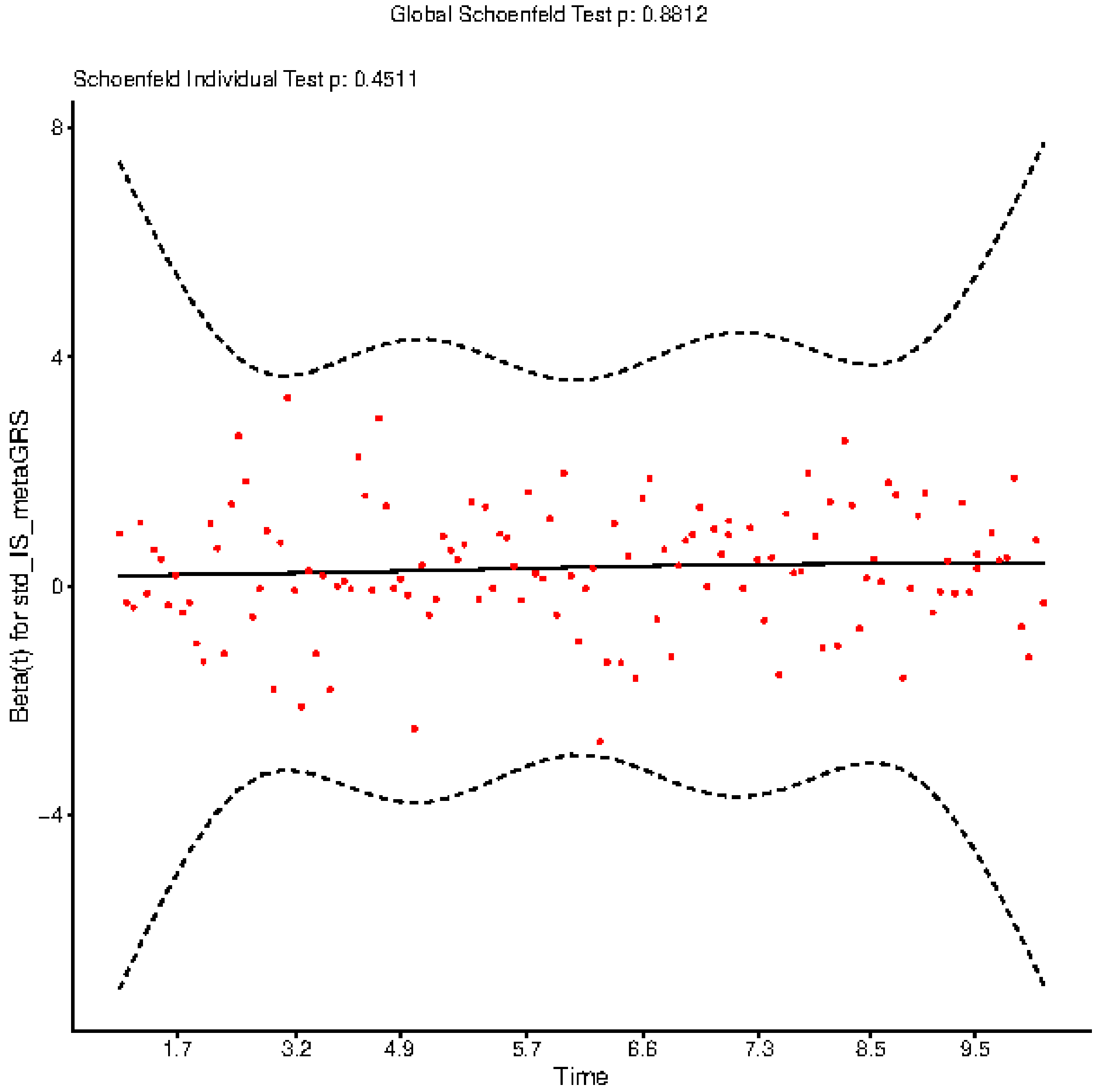

H: Schoenfeld residuals for the Cox proportional hazards model testing the association of the IS PRS with ASCVD in Black participants, adjusting for age, gender, and traditional risk factors.

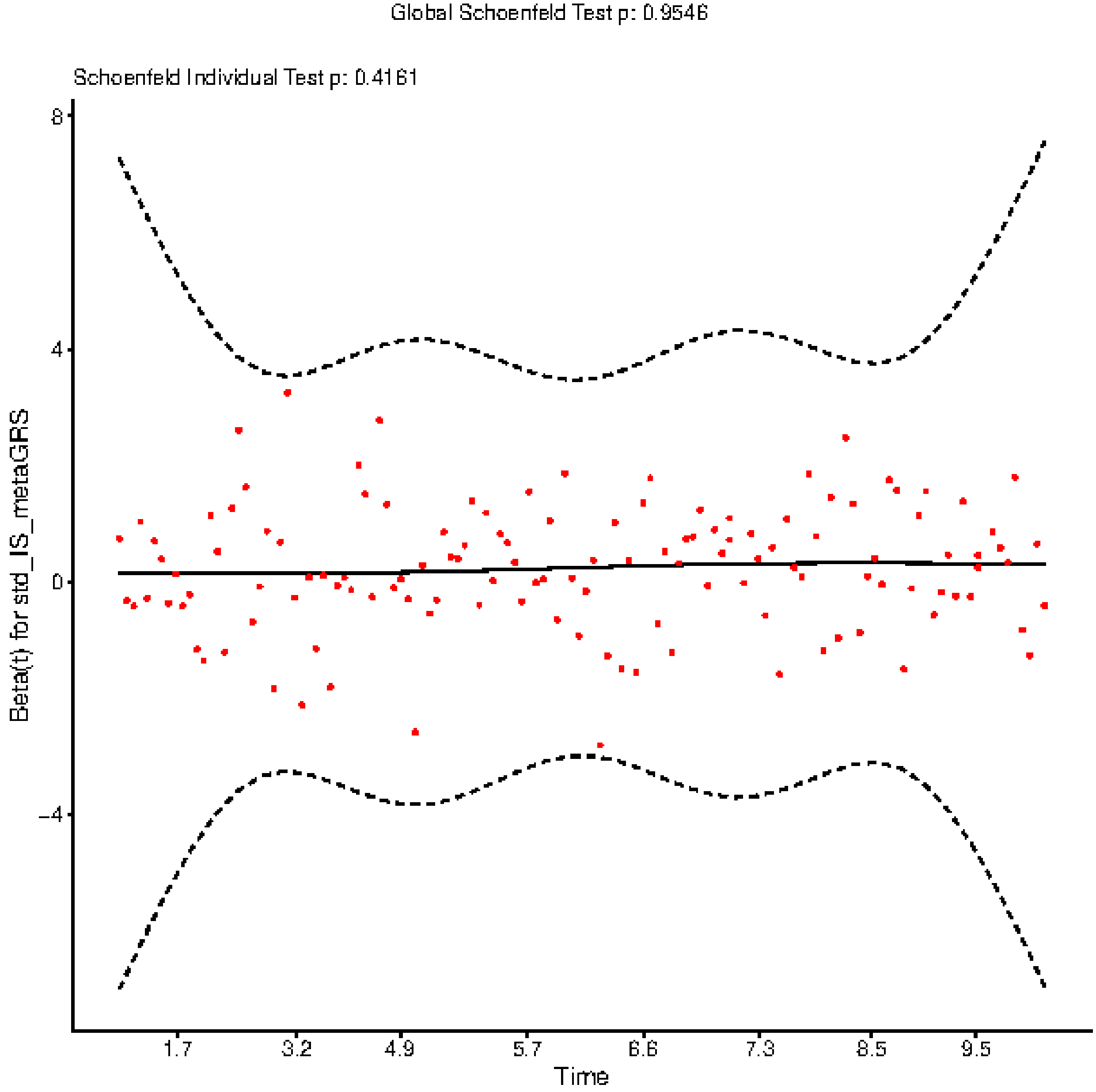

I: Schoenfeld residuals for the Cox proportional hazards model testing the association of the CHD PRS with CHD in White participants, adjusting for age and gender.

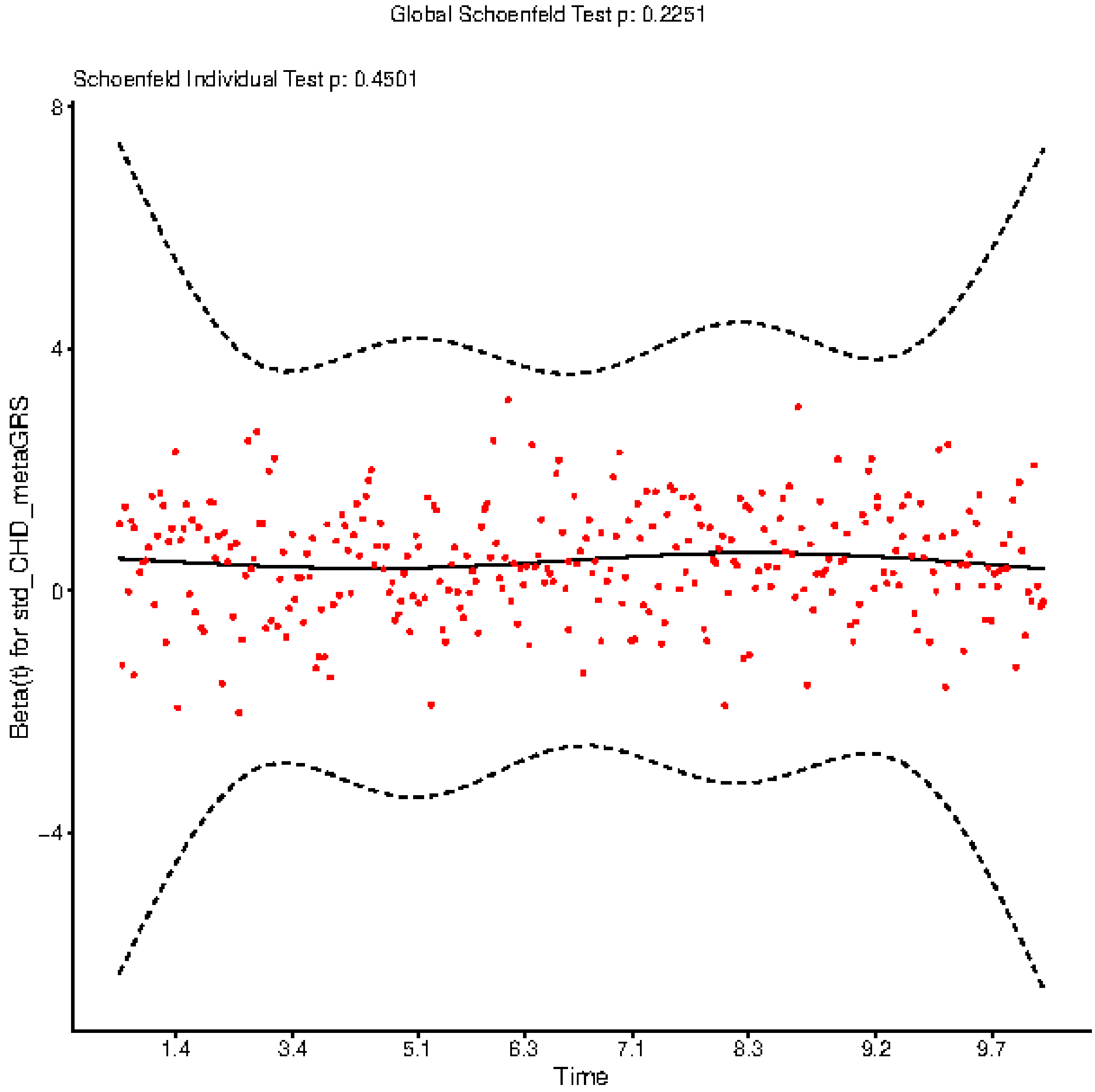

J: Schoenfeld residuals for the Cox proportional hazards model testing the association of the CHD PRS with CHD in White participants, adjusting for age, gender, and traditional risk factors.

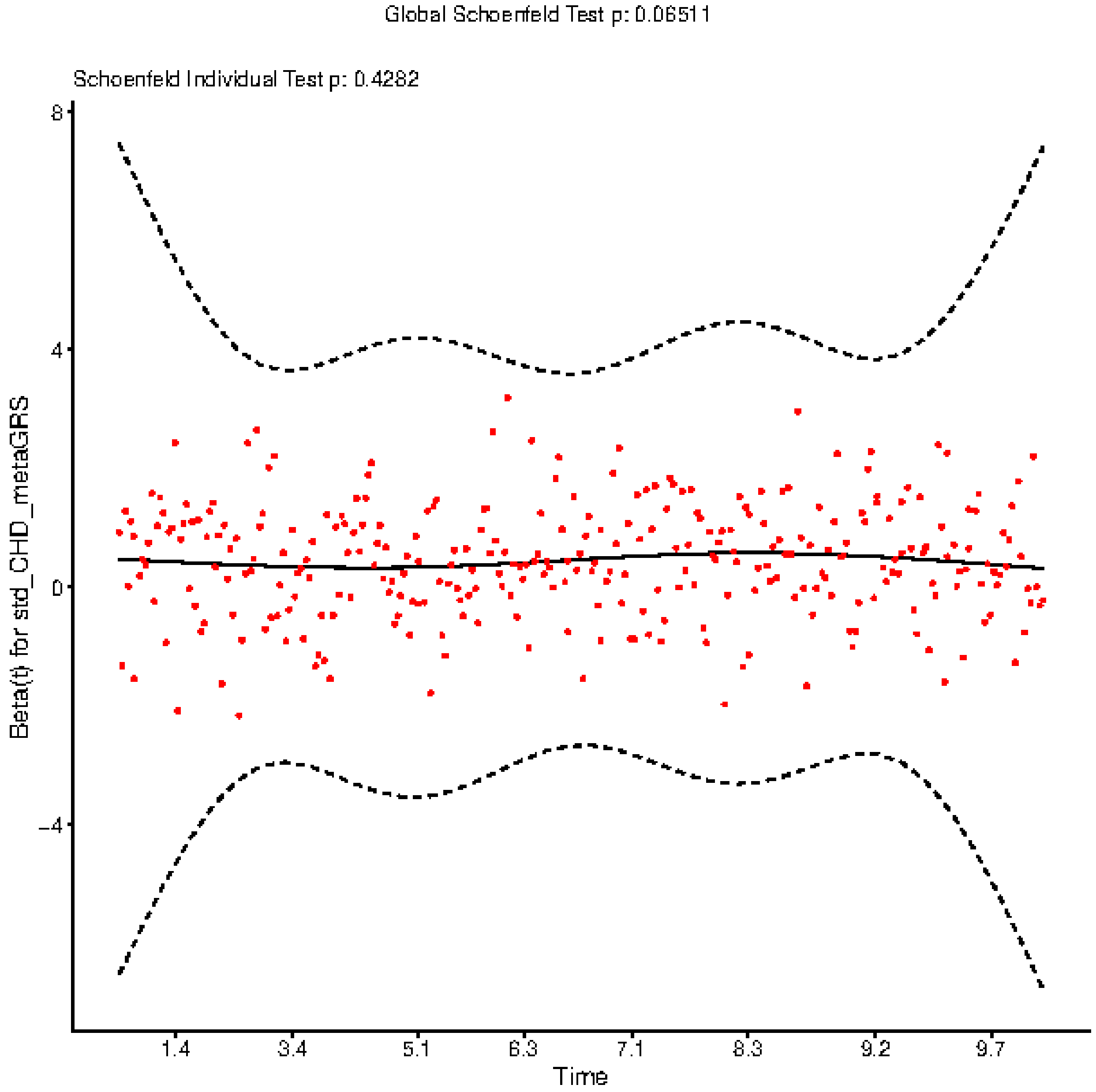

K: Schoenfeld residuals for the Cox proportional hazards model testing the association of the IS PRS with CHD in White participants, adjusting for age and gender.

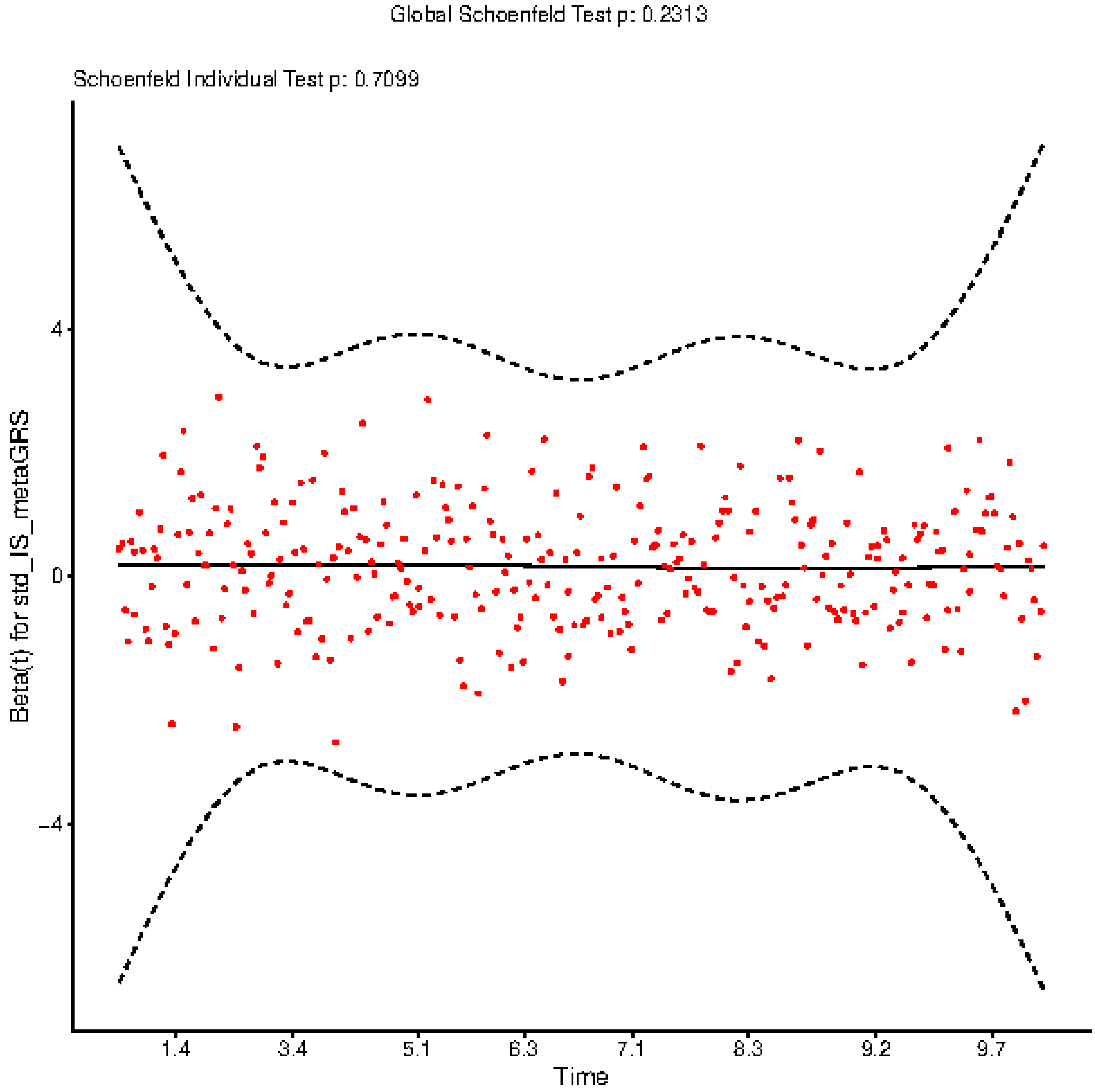

L: Schoenfeld residuals for the Cox proportional hazards model testing the association of the IS PRS with CHD in White participants, adjusting for age, gender, and traditional risk factors.

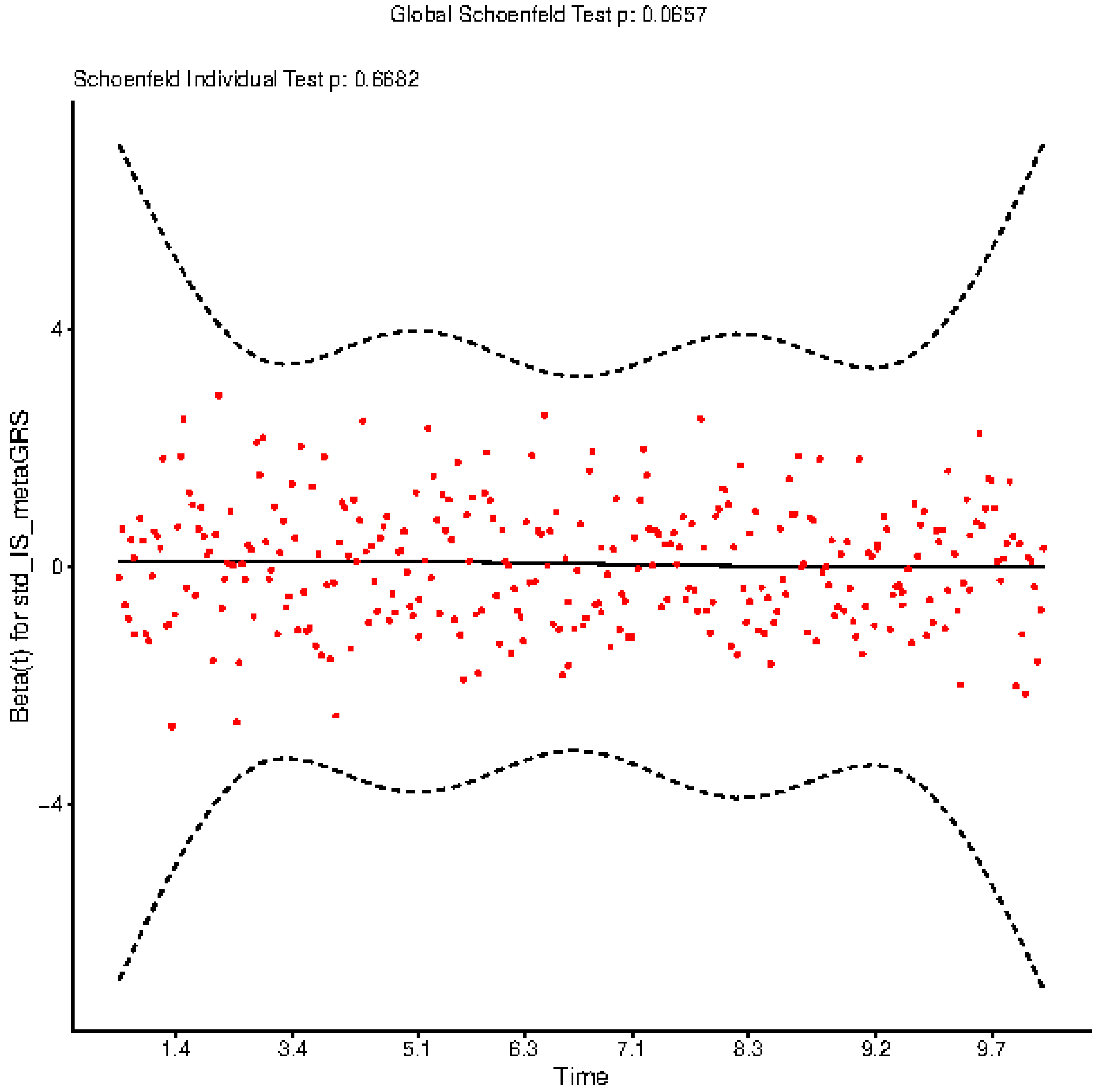

M: Schoenfeld residuals for the Cox proportional hazards model testing the association of the CHD PRS with CHD in Black participants, adjusting for age and gender.

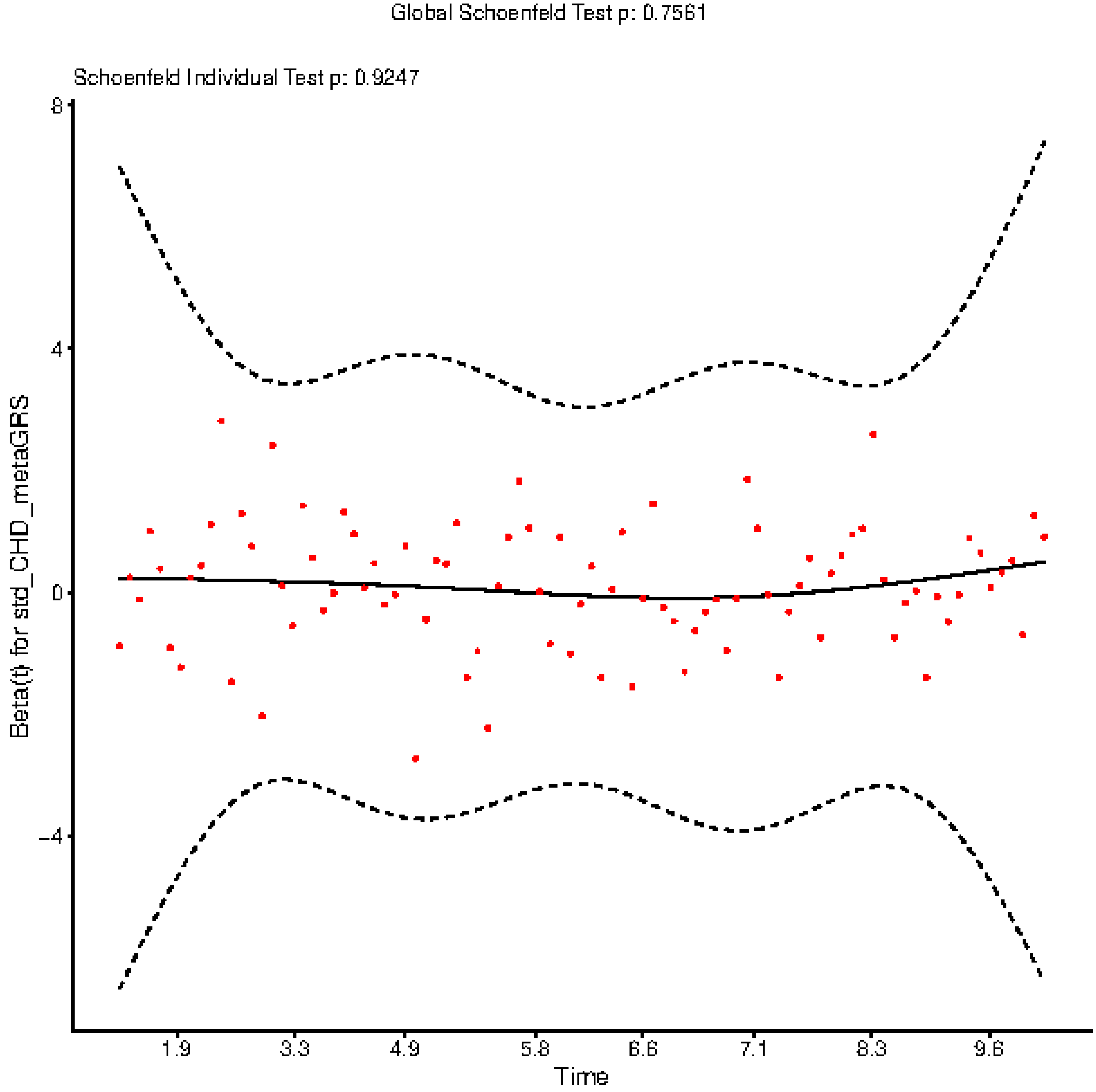

N: Schoenfeld residuals for the Cox proportional hazards model testing the association of the CHD PRS with CHD in Black participants, adjusting for age, gender, and traditional risk factors.

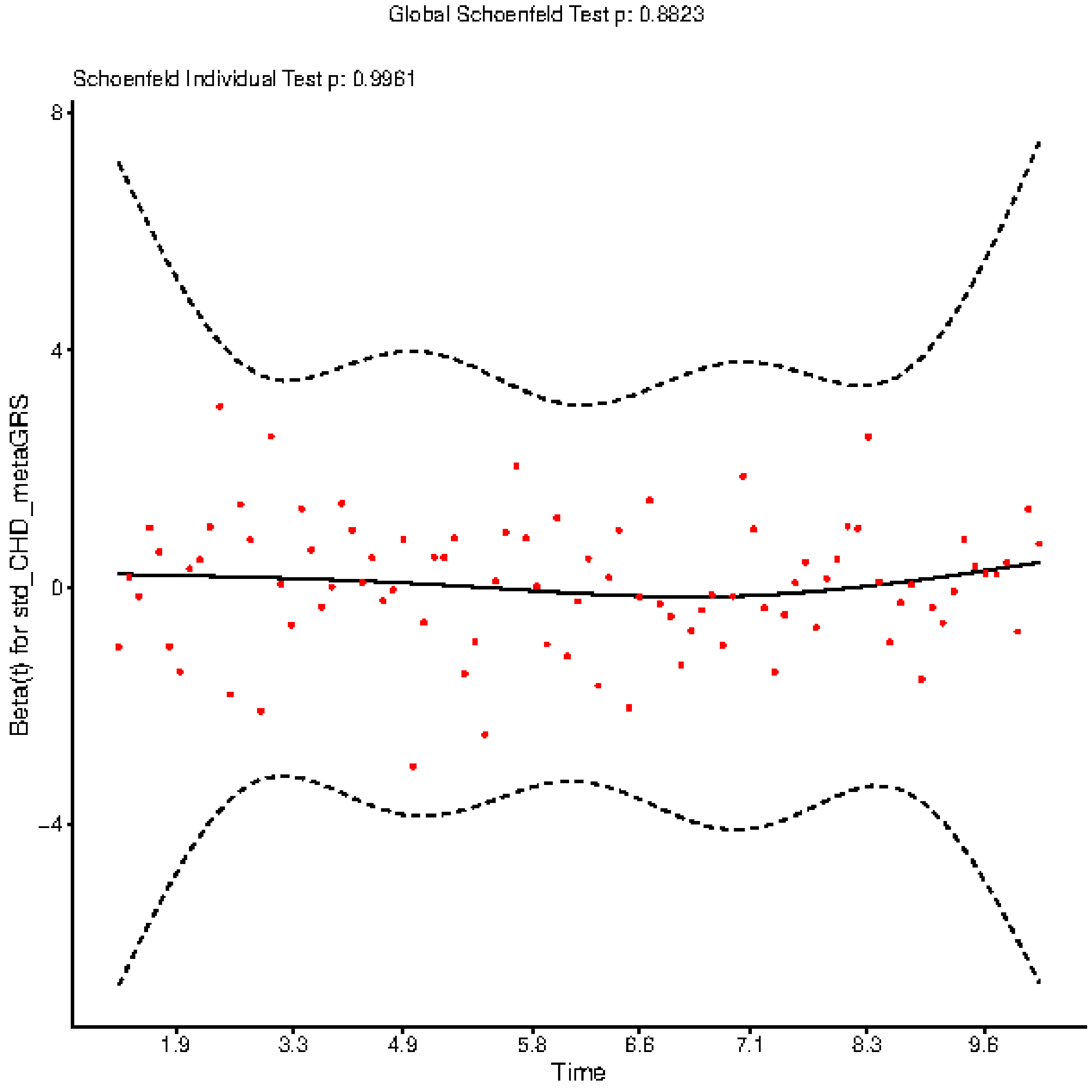

O: Schoenfeld residuals for the Cox proportional hazards model testing the association of the IS PRS with CHD in Black participants, adjusting for age and gender.

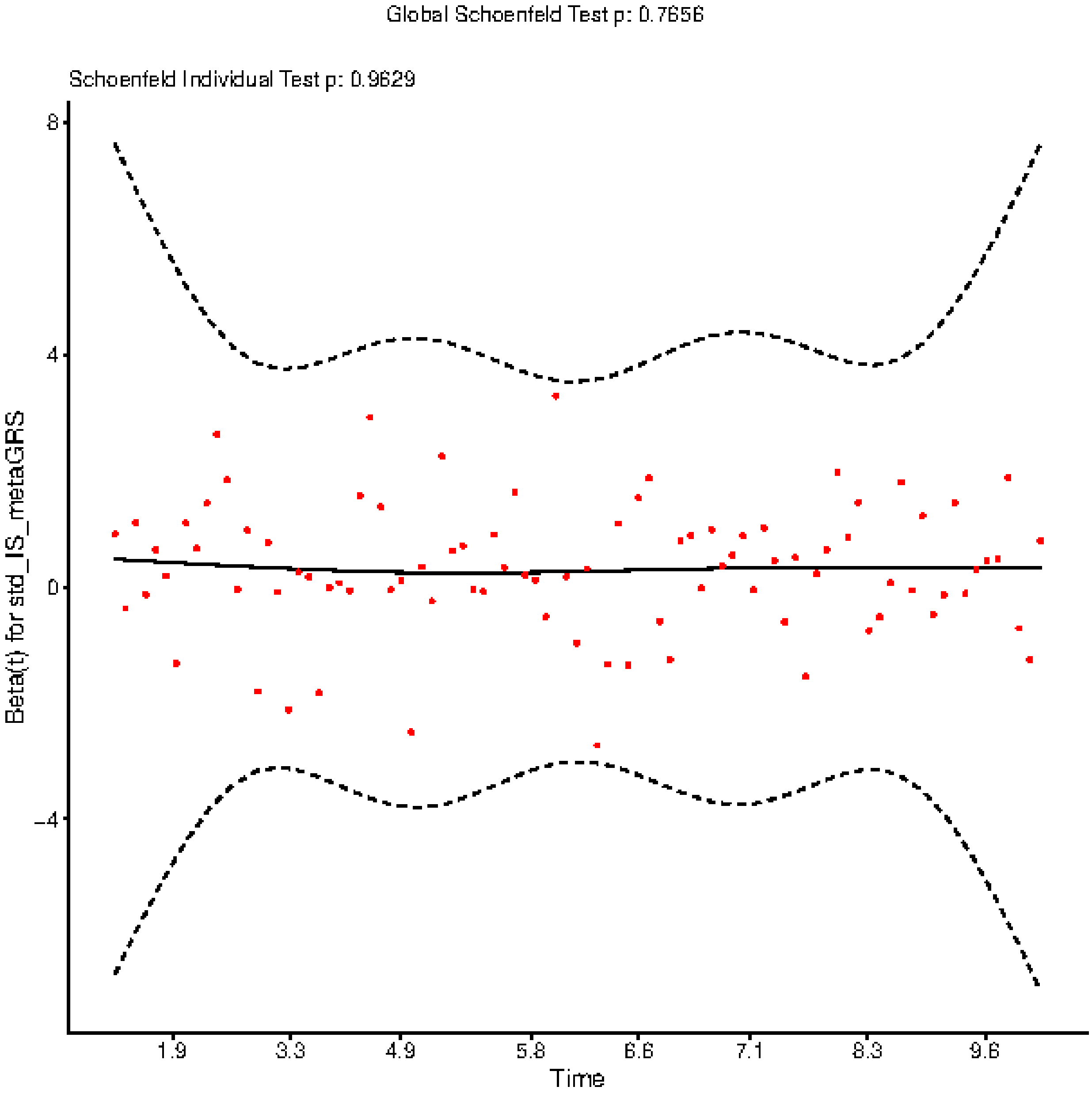

P: Schoenfeld residuals for the Cox proportional hazards model testing the association of the IS PRS with CHD in Black participants, adjusting for age, gender, and traditional risk factors.

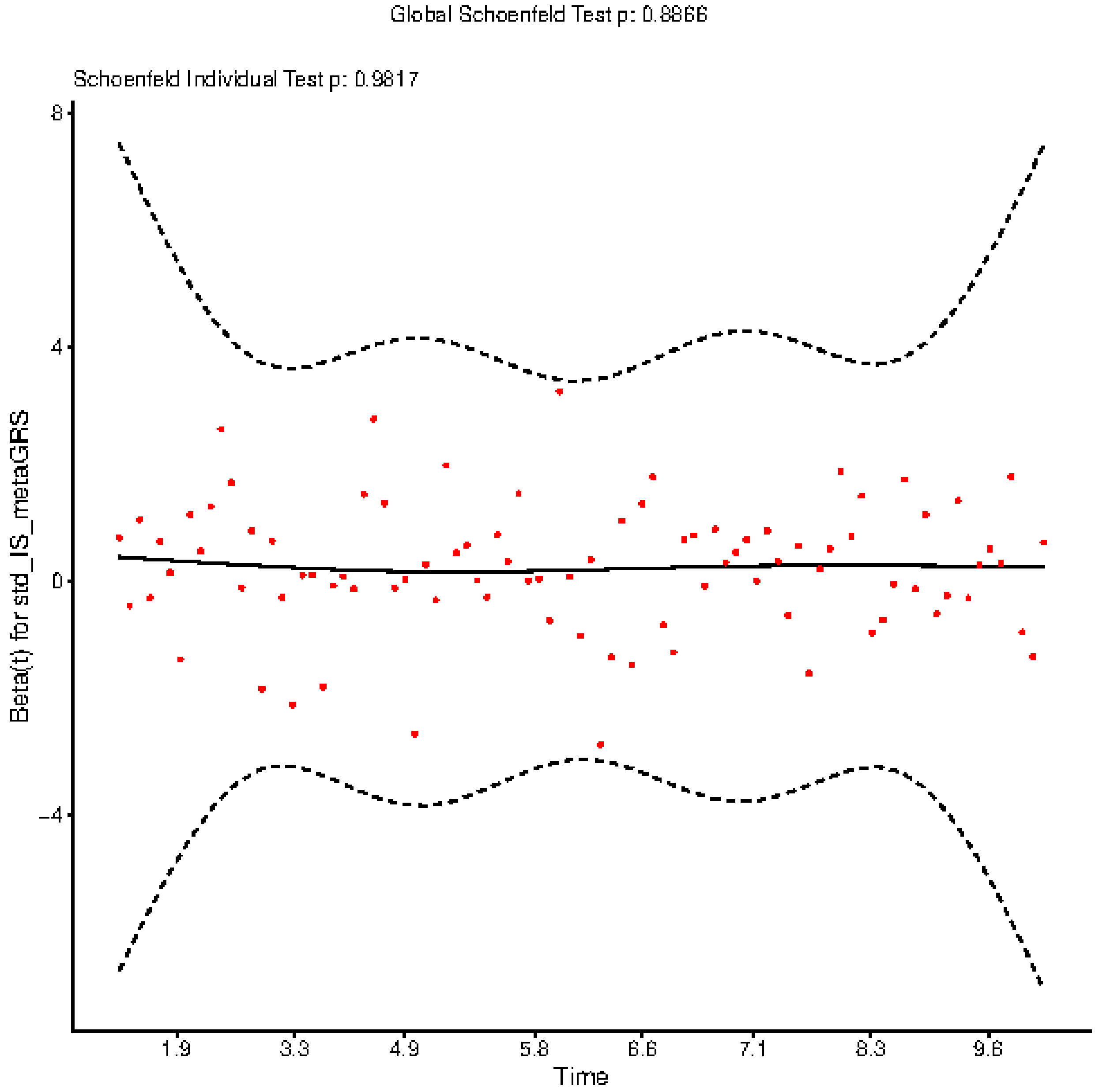

Q: Schoenfeld residuals for the Cox proportional hazards model testing the association of the CHD PRS with IS in White participants, adjusting for age and gender.

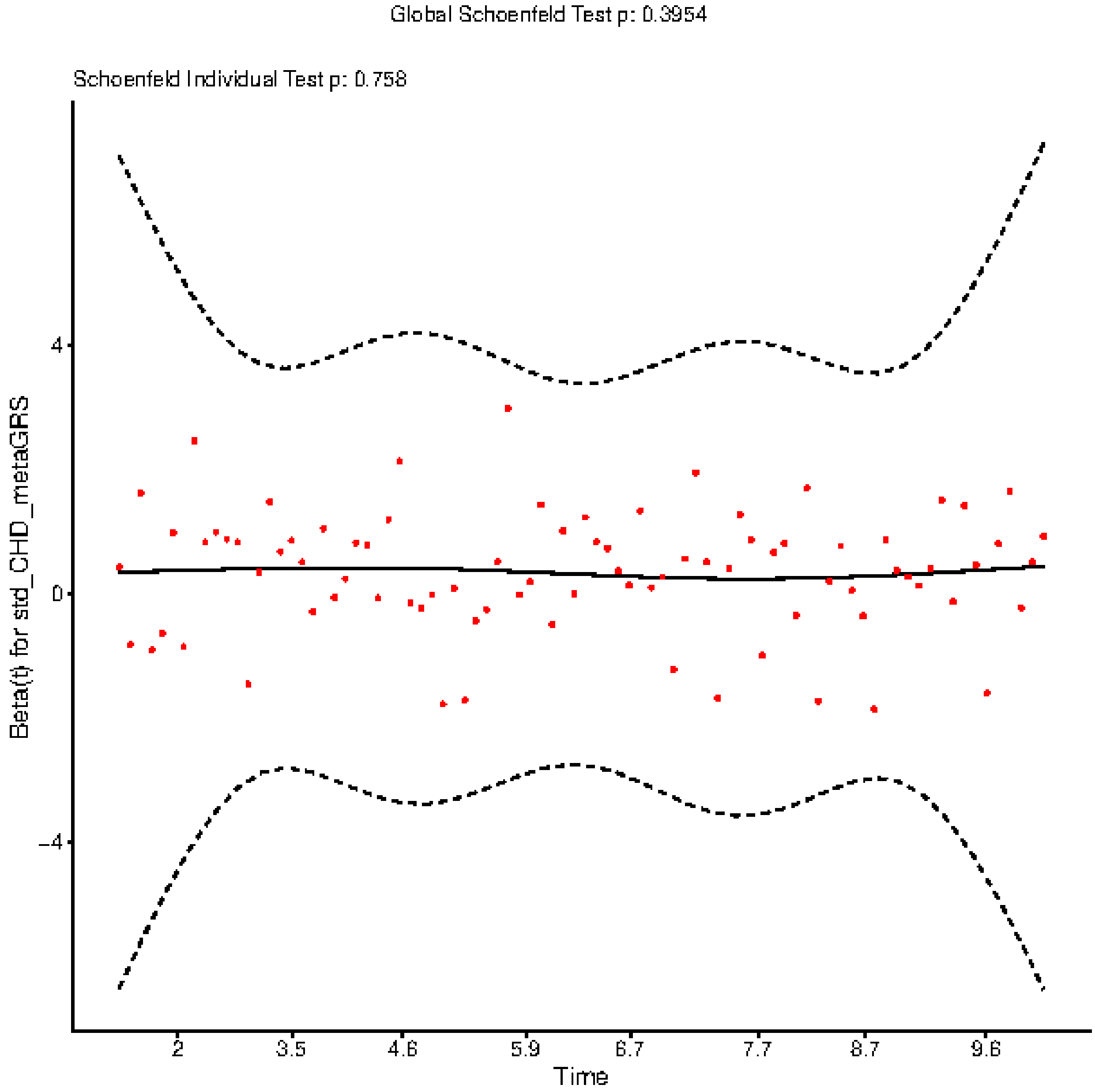

R: Schoenfeld residuals for the Cox proportional hazards model testing the association of the CHD PRS with IS in White participants, adjusting for age, gender, and traditional risk factors.

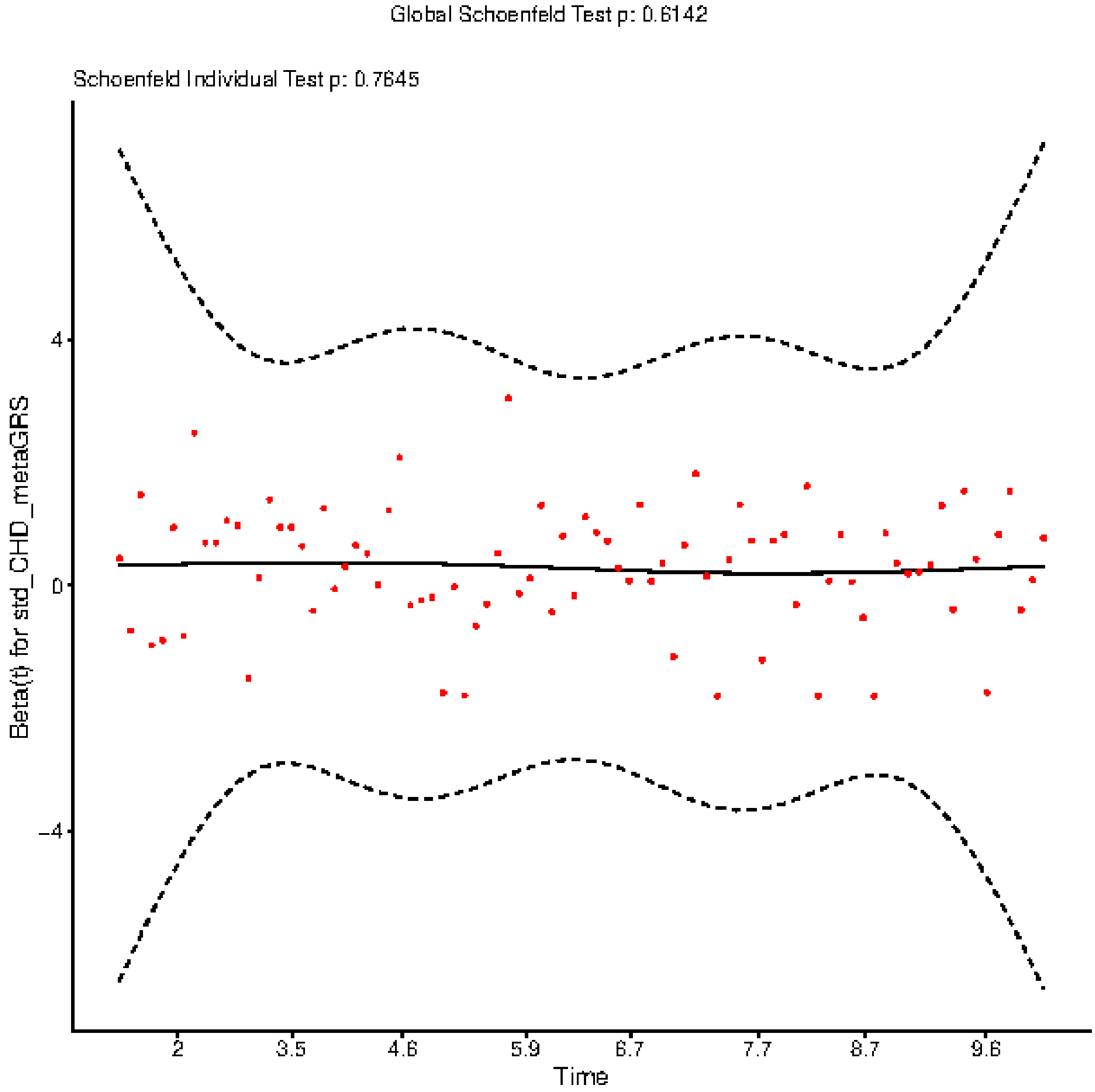

S: Schoenfeld residuals for the Cox proportional hazards model testing the association of the IS PRS with IS in White participants, adjusting for age and gender.

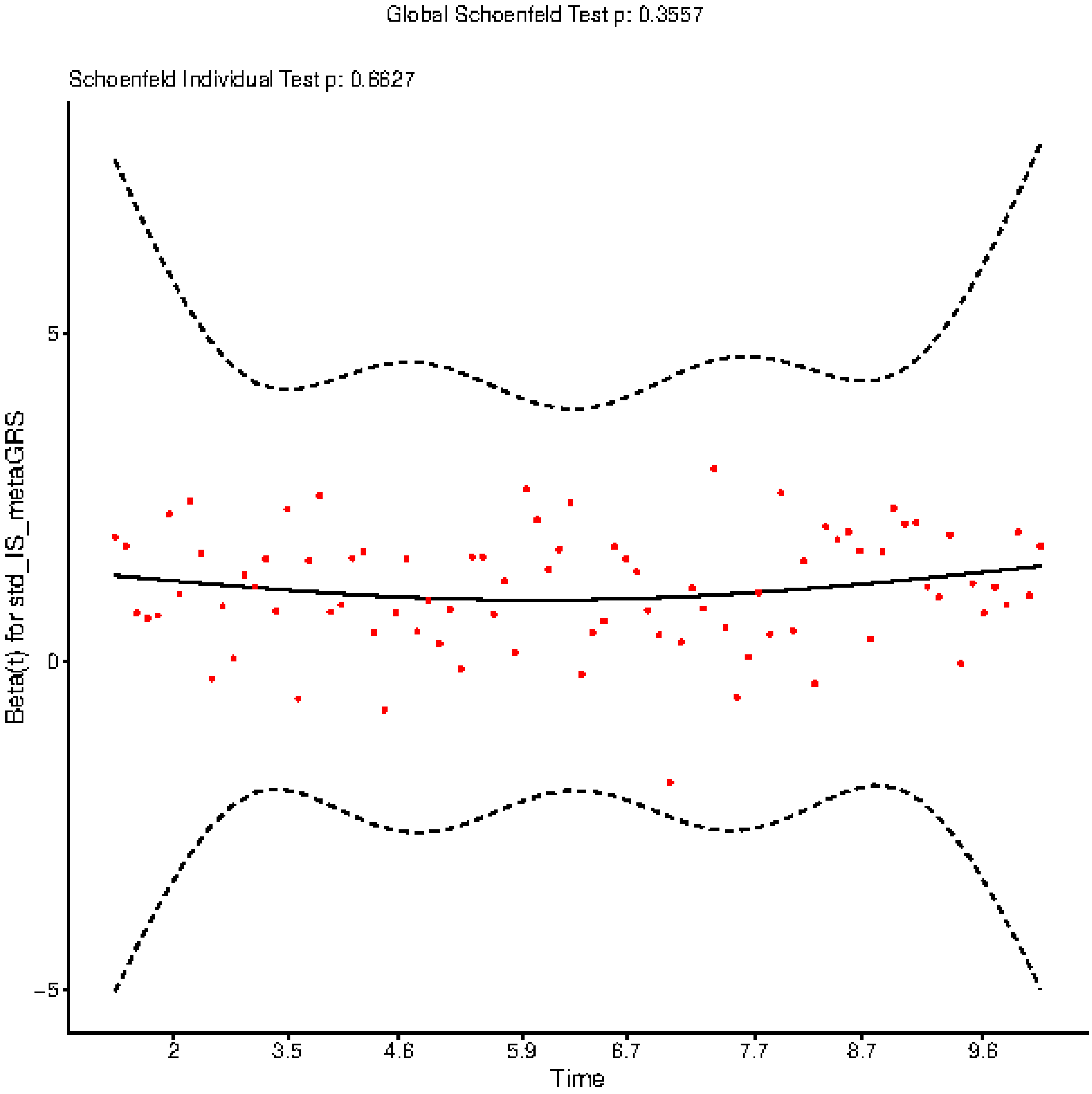

T: Schoenfeld residuals for the Cox proportional hazards model testing the association of the IS PRS with IS in White participants, adjusting for age, gender, and traditional risk factors.

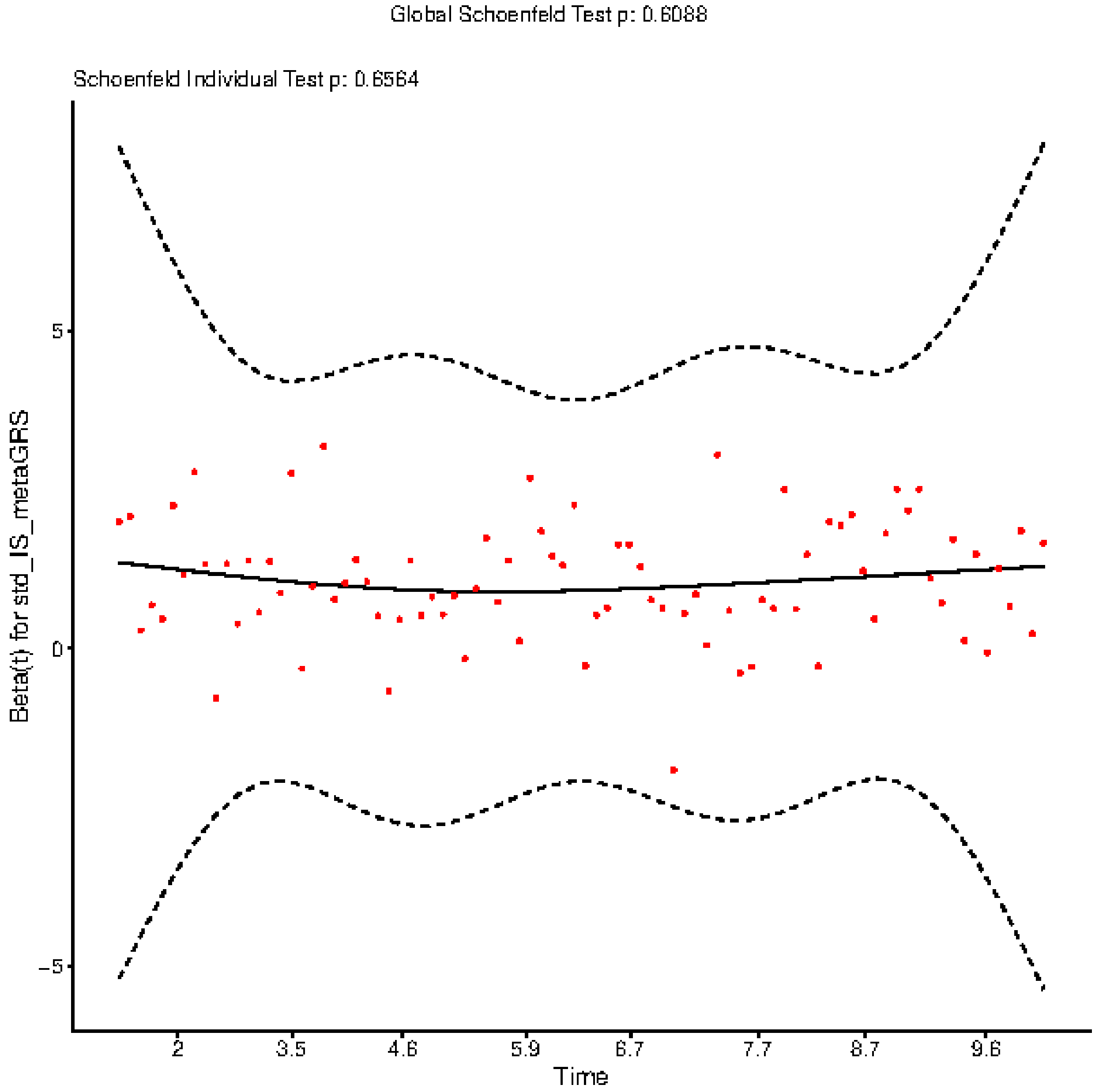

U: Schoenfeld residuals for the Cox proportional hazards model testing the association of the CHD PRS with IS in Black participants, adjusting for age and gender.

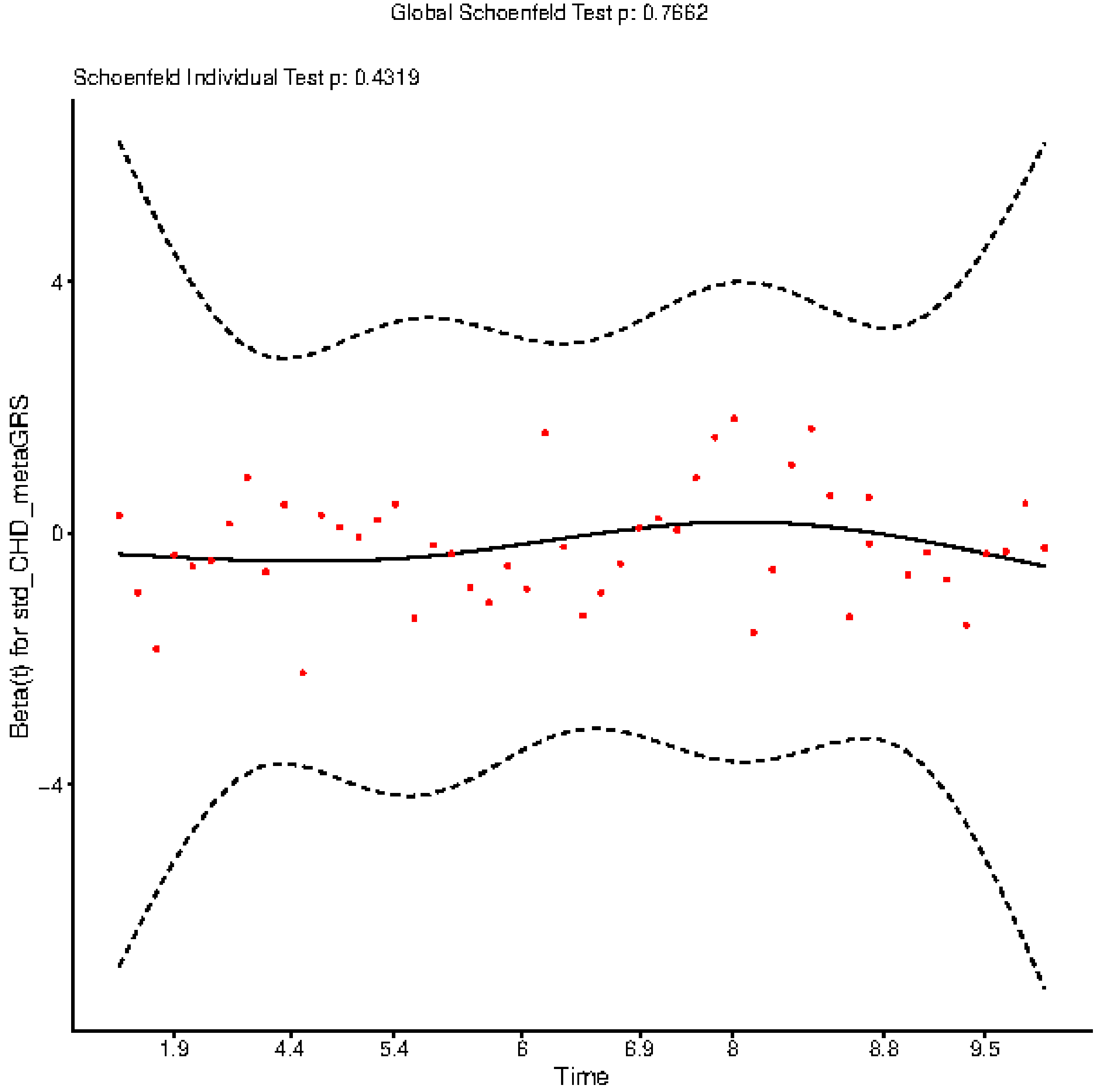

V: Schoenfeld residuals for the Cox proportional hazards model testing the association of the CHD PRS with IS in Black participants, adjusting for age, gender, and traditional risk factors.

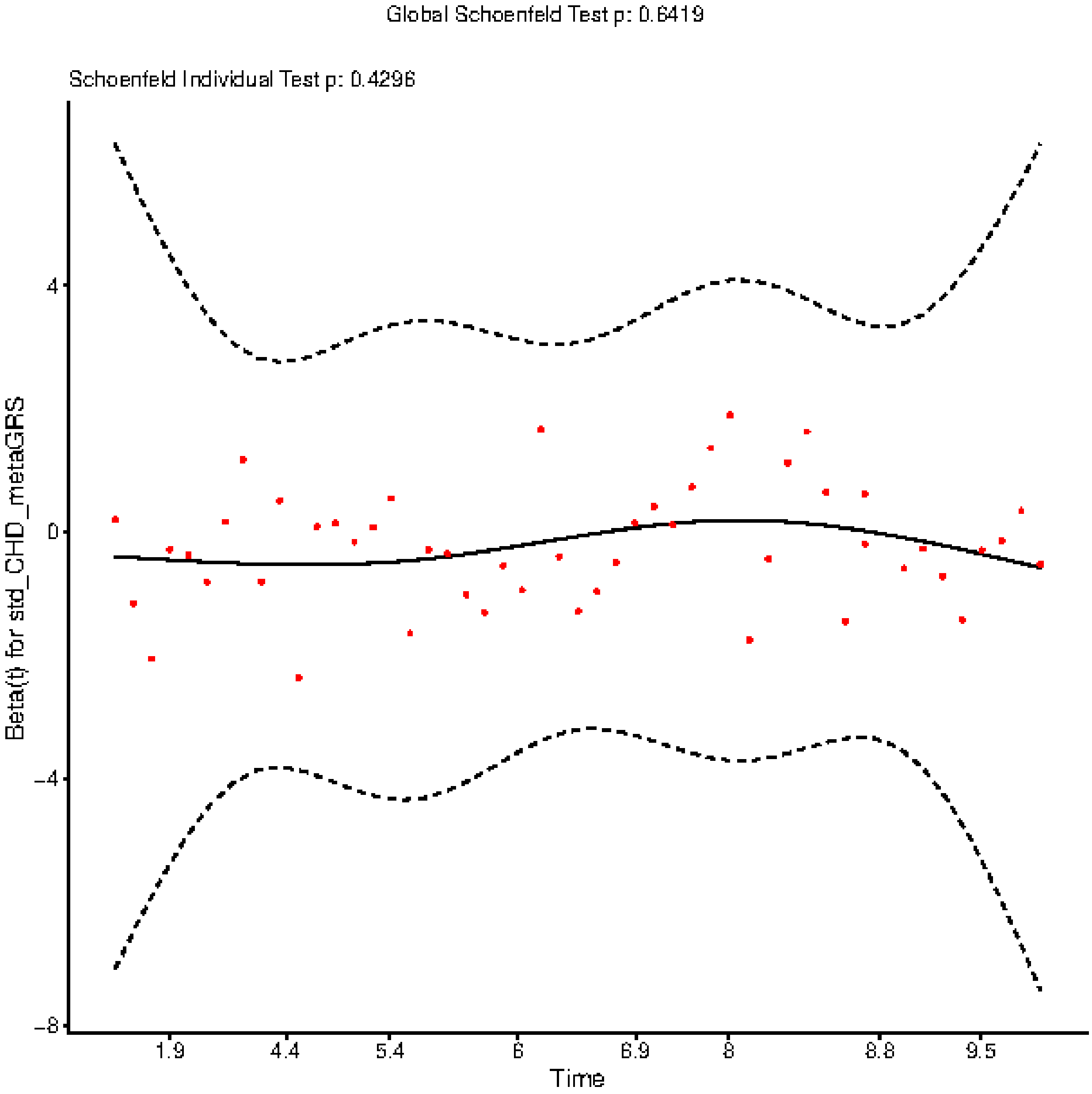

W: Schoenfeld residuals for the Cox proportional hazards model testing the association of the IS PRS with IS in Black participants, adjusting for age and gender.

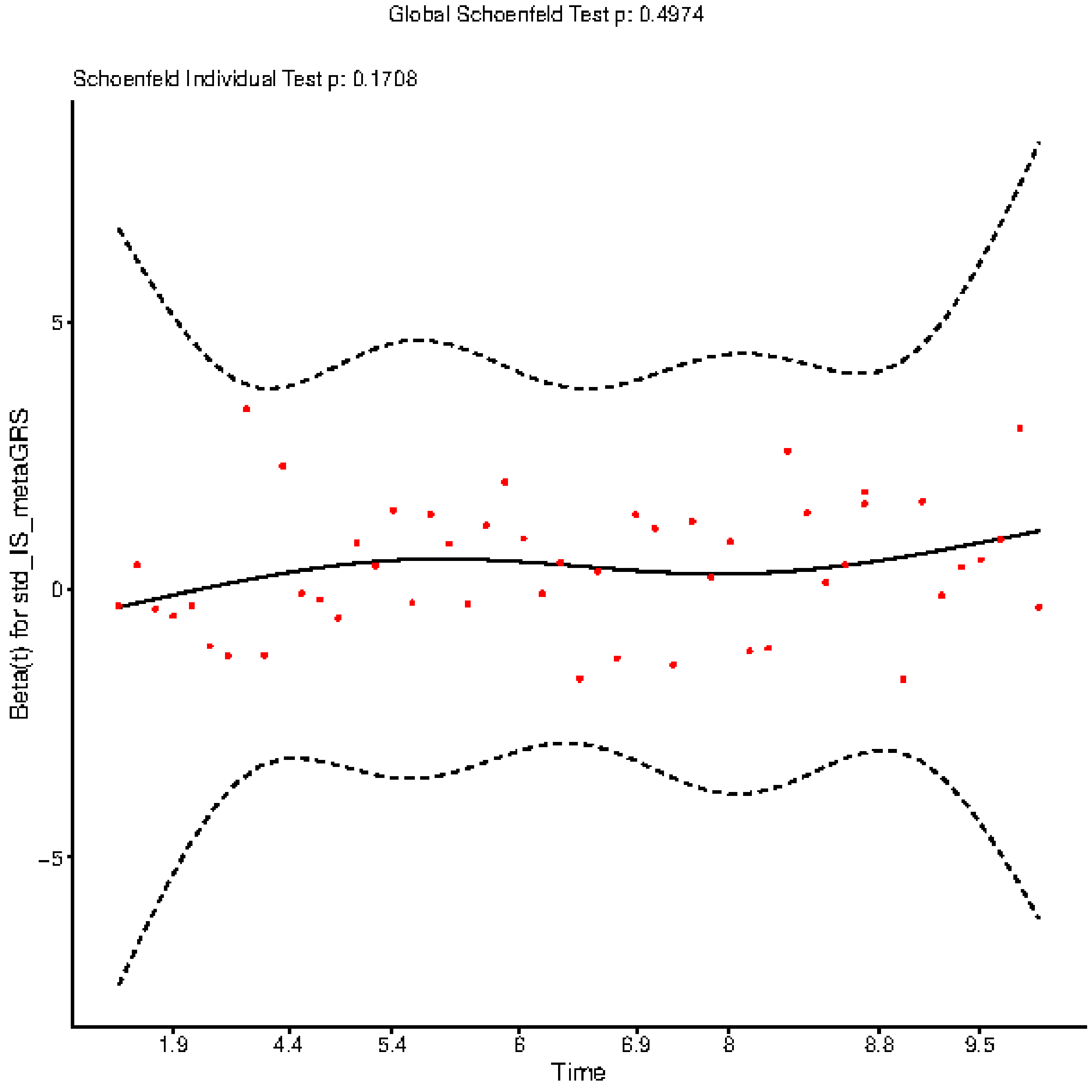

X: Schoenfeld residuals for the Cox proportional hazards model testing the association of the IS PRS with IS in Black participants, adjusting for age, gender, and traditional risk factors.

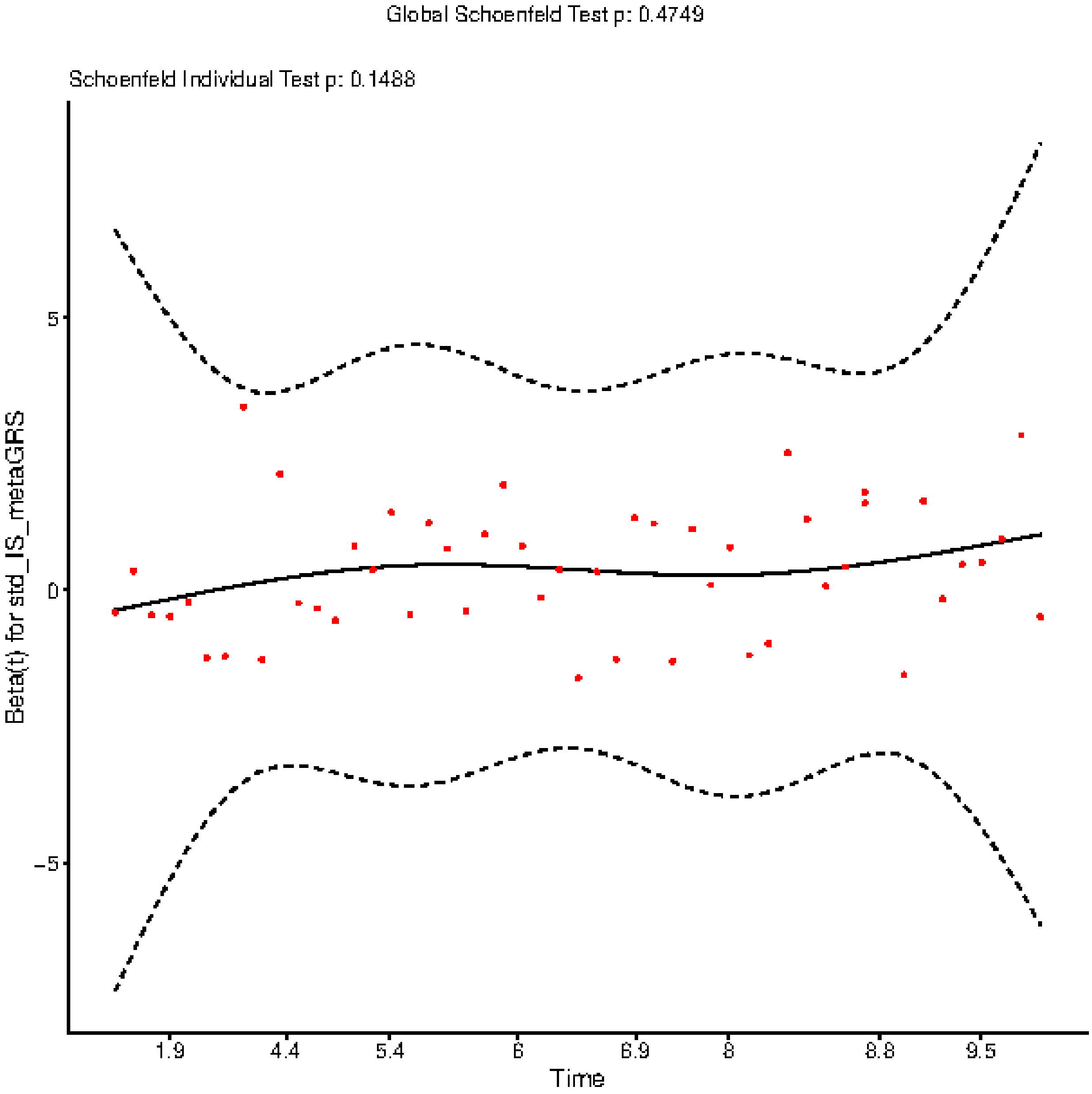

Supplement: S5 Fig — (PDF) [file pone.0285259.s005.pdf]
