# Supplementary material for: Coronary heart disease and ischemic stroke polygenic risk scores and atherosclerotic cardiovascular disease in a diverse, population-based cohort study
Source: PLoS One. 2023 Jun 16;18(6):e0285259. doi: 10.1371/journal.pone.0285259 (PMC10275447; doi:10.1371/journal.pone.0285259)

S6 Fig. Log-log plots for the Cox proportional hazards models testing the association of the CHD and IS PRS with ASCVD, CHD, and IS.

A: Log-log plot for the Cox proportional hazards model testing the association of the categorical CHD PRS with ASCVD in White participants.

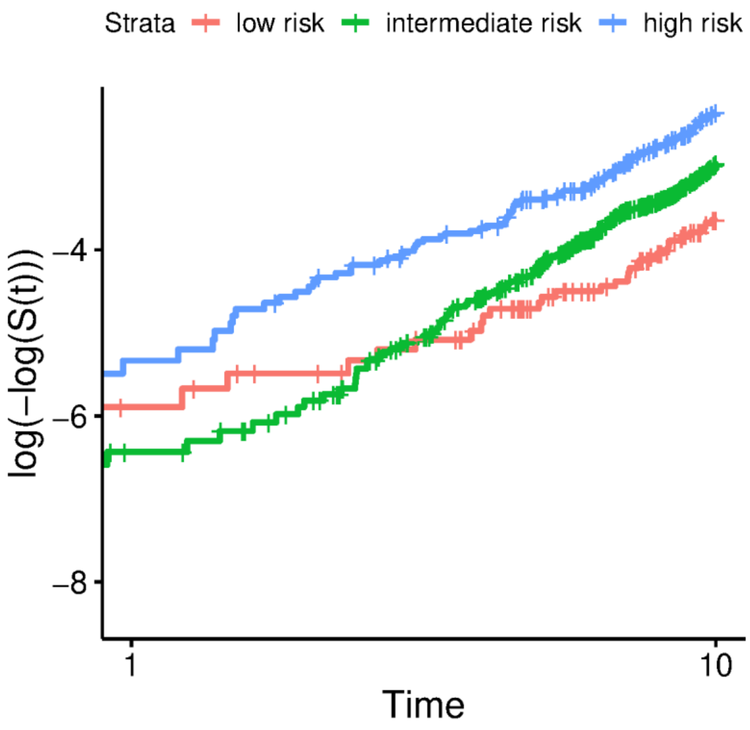

B: Log-log plot for the Cox proportional hazards model testing the association of the categorical IS PRS with ASCVD in White participants.

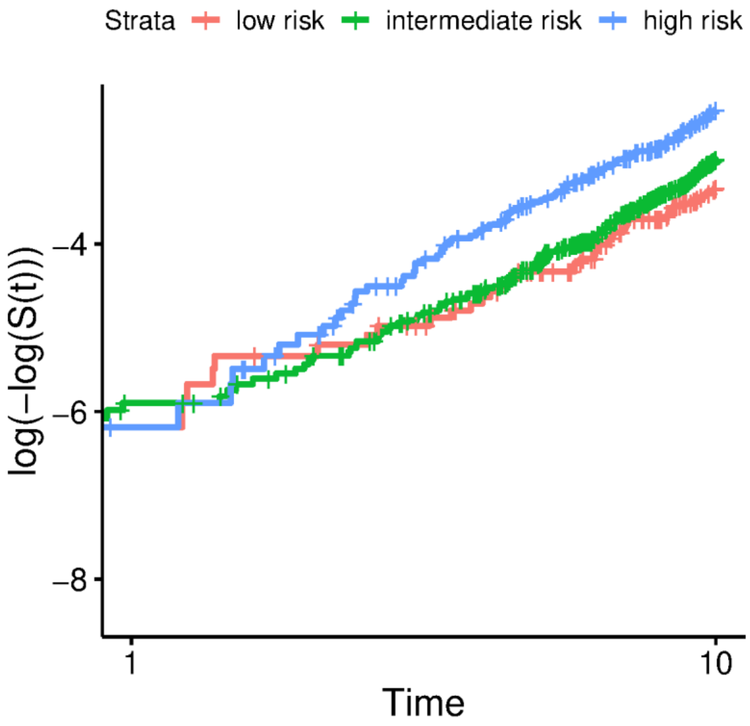

C: Log-log plot for the Cox proportional hazards model testing the association of the categorical CHD PRS with ASCVD in Black participants.

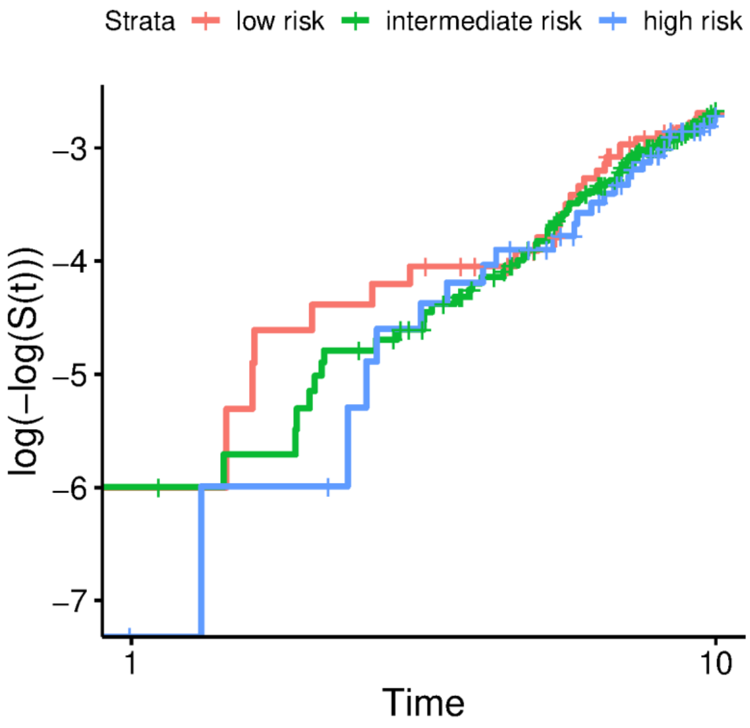

D: Log-log plot for the Cox proportional hazards model testing the association of the categorical IS PRS with ASCVD in Black participants.

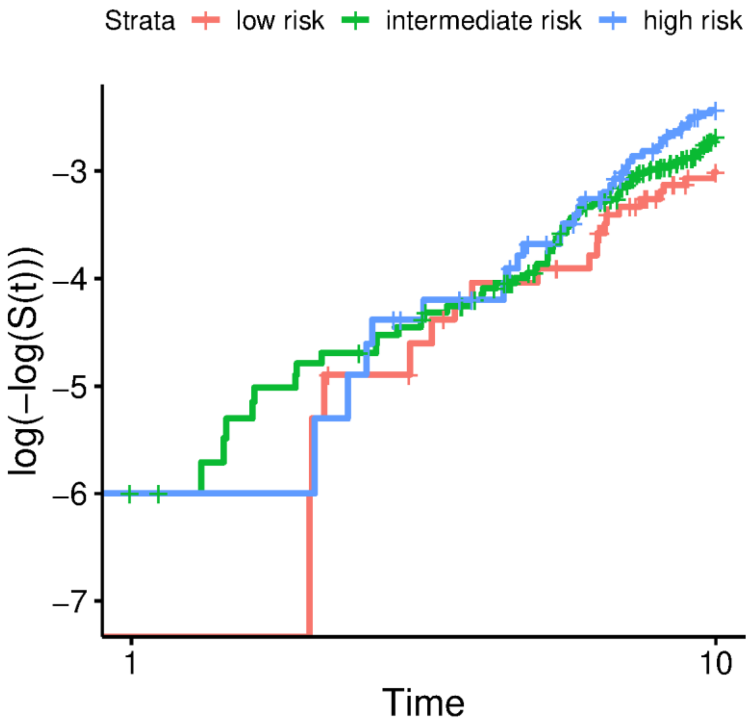

E: Log-log plot for the Cox proportional hazards model testing the association of the categorical CHD PRS with CHD in White participants.

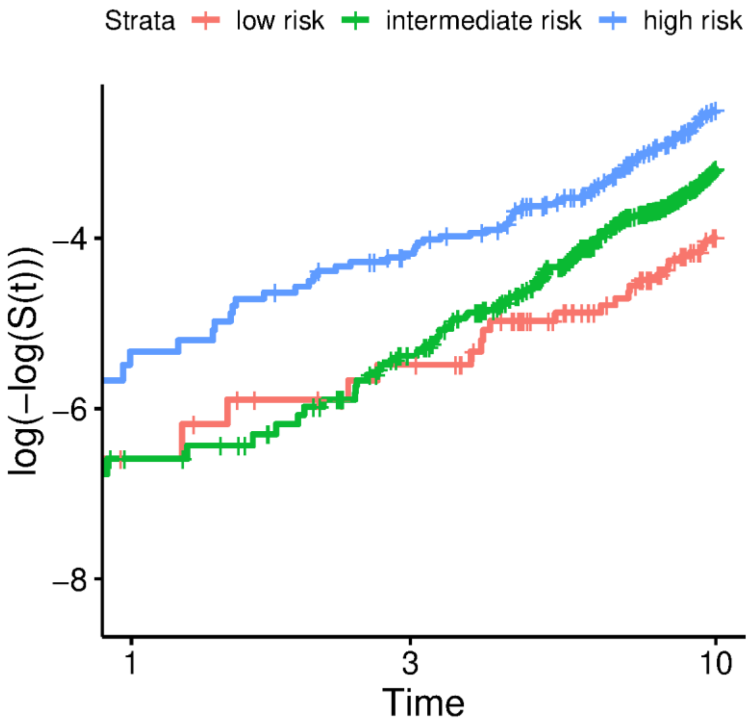

F: Log-log plot for the Cox proportional hazards model testing the association of the categorical IS PRS with CHD in White participants.

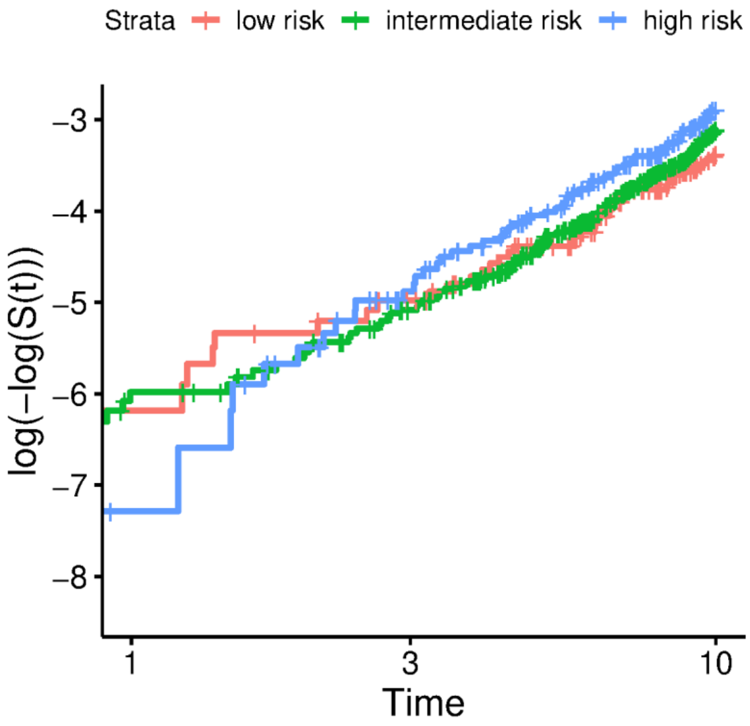

G: Log-log plot for the Cox proportional hazards model testing the association of the categorical CHD PRS with CHD in Black participants.

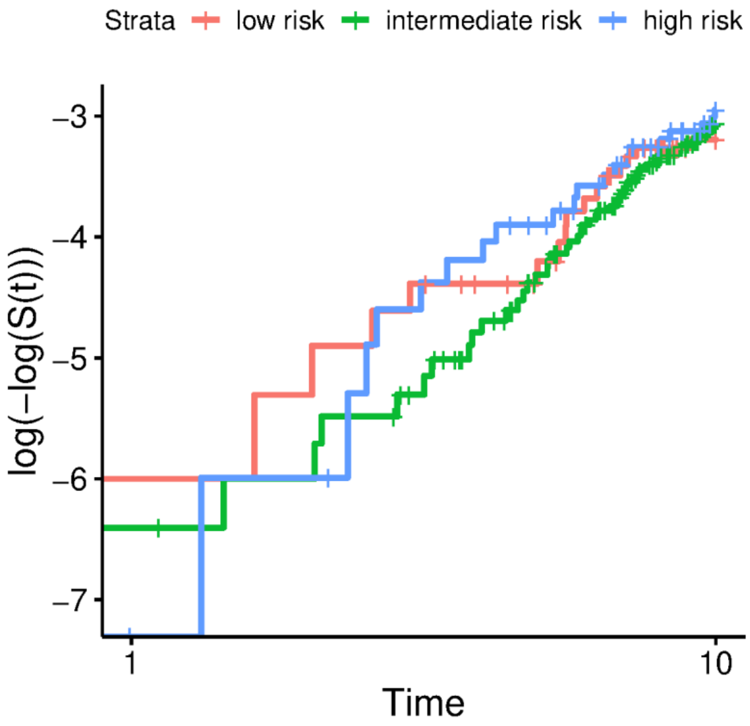

H: Log-log plot for the Cox proportional hazards model testing the association of the categorical IS PRS with CHD in Black participants.

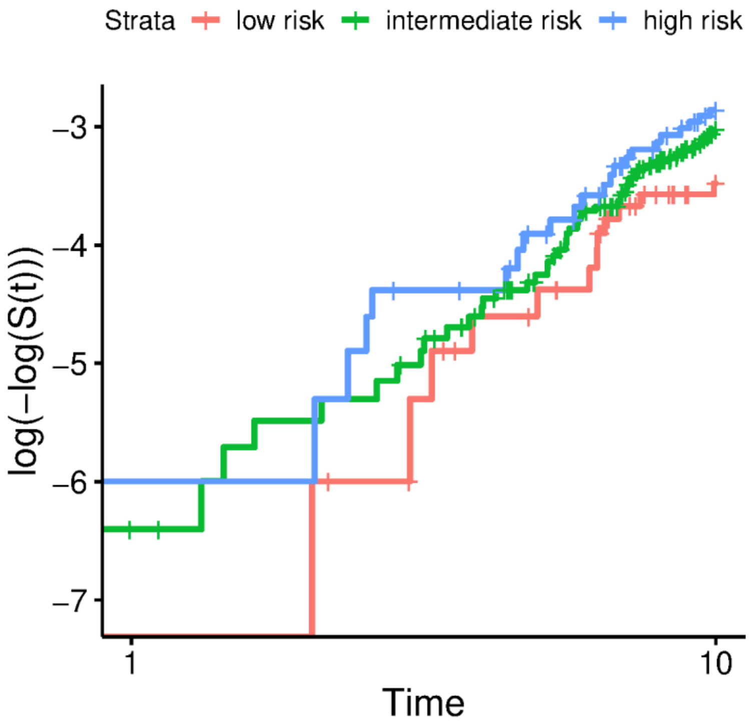

I: Log-log plot for the Cox proportional hazards model testing the association of the categorical CHD PRS with IS in White participants.

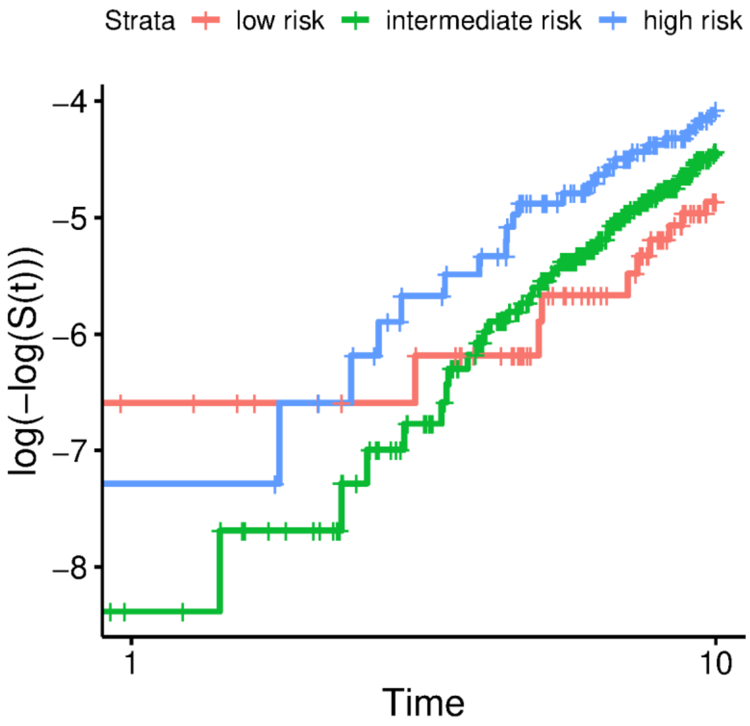

J: Log-log plot for the Cox proportional hazards model testing the association of the categorical IS PRS with IS in White participants.

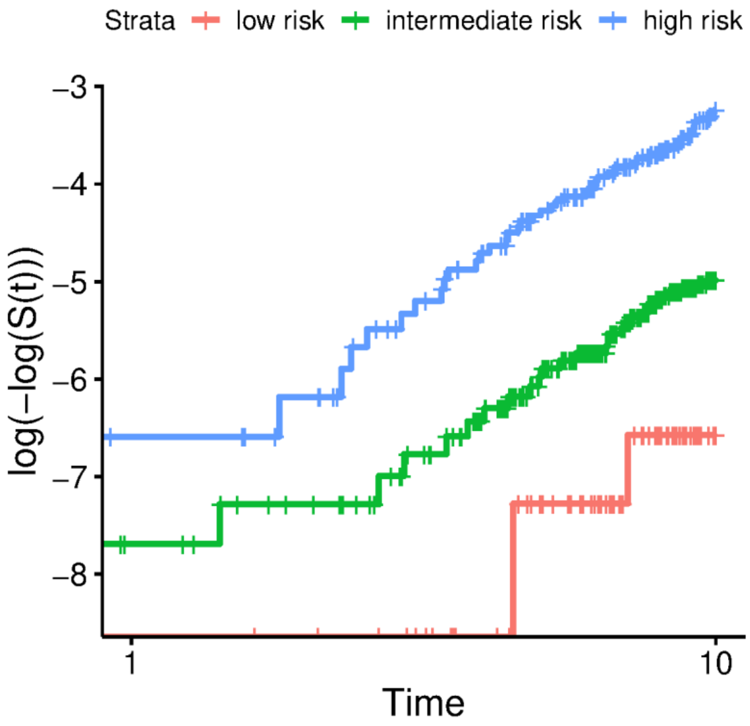

K: Log-log plot for the Cox proportional hazards model testing the association of the categorical CHD PRS with IS in Black participants.

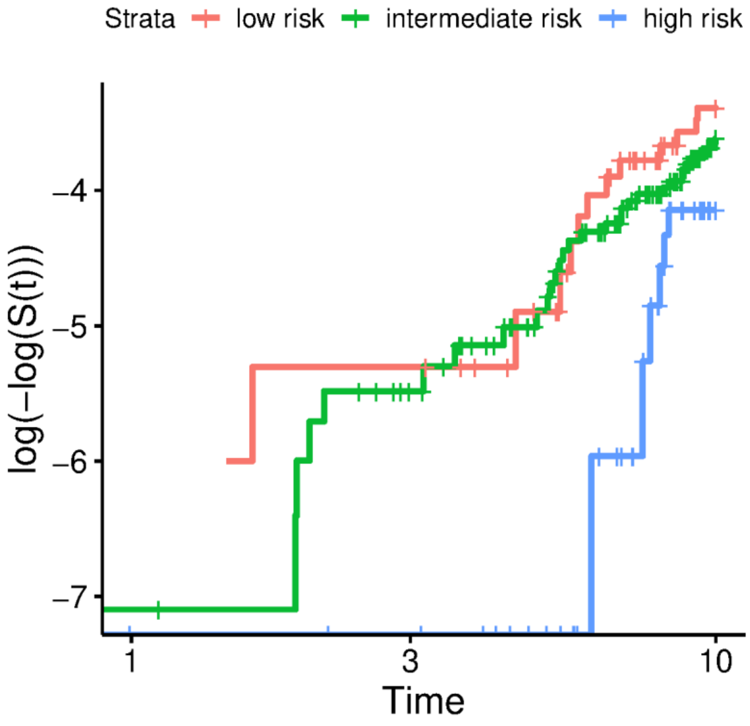

L: Log-log plot for the Cox proportional hazards model testing the association of the categorical IS PRS with IS in Black participants.

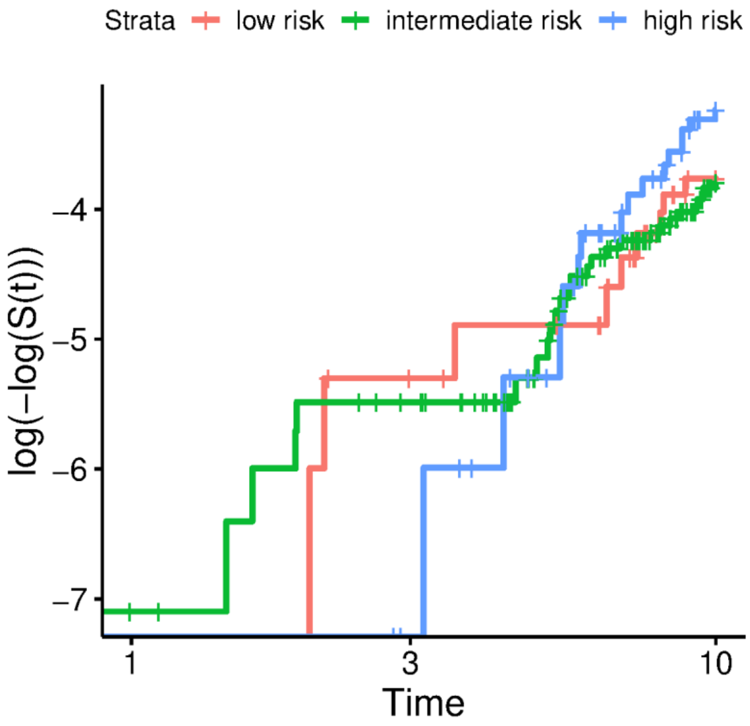

Supplement: S6 Fig — (PDF) [file pone.0285259.s006.pdf]
