# Supplementary material for: Coronary heart disease and ischemic stroke polygenic risk scores and atherosclerotic cardiovascular disease in a diverse, population-based cohort study
Source: PLoS One. 2023 Jun 16;18(6):e0285259. doi: 10.1371/journal.pone.0285259 (PMC10275447; doi:10.1371/journal.pone.0285259)
Supplement: S1 Table — (PDF) [file pone.0285259.s007.pdf]

S1 Table. Association of categorical PRS with time-to-ASCVD, time-to-CHD, and time-to-IS in the first 10 years of follow-up.

	**atherosclerotic cardiovascular disease**			
	**Adujusted for age and sex**		**Adjusted for traditional risk factors**	
	**HR (95% CI)**	**P-value**	**HR (95% CI)**	**P-value**
	European Americans (Ncases=380, N=7286)			
low CHD PRS	0.48 (0.34, 0.68)	**3.45E-05**	0.52 (0.36, 0.73)	**2.21E-04**
high CHD PRS	1.95 (1.57, 2.43)	**1.84E-09**	1.78 (1.43, 2.22)	**2.29E-07**
low IS PRS	0.67 (0.49, 0.92)	**0.0121**	0.78 (0.57, 1.07)	0.121
high IS PRS	1.77 (1.42, 2.22)	**5.28E-07**	1.54 (1.23, 1.93)	**1.80E-04**
	African Americans (Ncases=130, N=2016)			
low CHD PRS	0.97 (0.63, 1.52)	0.909	1.13 (0.72, 1.77)	0.593
high CHD PRS	1.02 (0.65, 1.59)	0.947	1.01 (0.64, 1.58)	0.982
low IS PRS	0.72 (0.44, 1.19)	0.200	0.80 (0.48, 1.32)	0.384
high IS PRS	1.36 (0.90, 2.04)	0.144	1.28 (0.85, 1.93)	0.233
	**coronary heart disease**			
	**Adujusted for age and sex**		**Adjusted for traditional risk factors**	
	**HR (95% CI)**	**P-value**	**HR (95% CI)**	**P-value**
	European Americans (Ncases=309, N=7286)			
low CHD PRS	0.43 (0.28, 0.65)	**5.87E-05**	0.46 (0.30, 0.69)	**1.94E-04**
high CHD PRS	2.05 (1.61, 2.60)	**4.12E-09**	1.90 (1.50, 2.42)	**1.40E-07**
low IS PRS	0.78 (0.57, 1.08)	0.134	0.87 (0.63, 1.20)	0.383
high IS PRS	1.16 (0.89, 1.52)	0.281	1.04 (0.79, 1.36)	0.783
	African Americans (Ncases=90, N=2016)			
low CHD PRS	0.88 (0.50, 1.54)	0.652	1.00 (0.57, 1.75)	0.998
high CHD PRS	1.19 (0.71, 2.00)	0.498	1.19 (0.71, 1.99)	0.518
low IS PRS	0.64 (0.34, 1.20)	0.164	0.70 (0.38, 1.31)	0.268
high IS PRS	1.23 (0.75, 2.02)	0.405	1.19 (0.73, 1.96)	0.481
	**ischemic stroke**			
	**Adujusted for age and sex**		**Adjusted for traditional risk factors**	
	**HR (95% CI)**	**P-value**	**HR (95% CI)**	**P-value**
	European Americans (Ncases=85, N=7286)			
low CHD PRS	0.61 (0.32, 1.17)	0.139	0.66 (0.34, 1.28)	0.219
high CHD PRS	1.51 (0.93, 2.46)	0.0973	1.38 (0.85, 2.26)	0.193
low IS PRS	0.19 (0.04, 0.79)	**0.0223**	0.21 (0.05, 0.87)	**0.0310**
high IS PRS	5.68 (3.61, 8.93)	**5.97E-14**	5.07 (3.21, 8.03)	**4.06E-12**

		African Americans (Ncases=50, N=2016)		
low CHD PRS	1.24 (0.65, 2.38)	0.510	1.40 (0.73, 2.70)	0.311
high CHD PRS	0.63 (0.26, 1.51)	0.296	0.63 (0.26, 1.53)	0.310
low IS PRS	1.02 (0.48, 2.17)	0.966	1.12 (0.52, 2.40)	0.780
high IS PRS	1.87 (0.99, 3.53)	0.0552	1.77 (0.94, 3.34)	0.0788
