# Supplementary material for: Coronary heart disease and ischemic stroke polygenic risk scores and atherosclerotic cardiovascular disease in a diverse, population-based cohort study
Source: PLoS One. 2023 Jun 16;18(6):e0285259. doi: 10.1371/journal.pone.0285259 (PMC10275447; doi:10.1371/journal.pone.0285259)
Supplement: S2 Table — (PDF) [file pone.0285259.s008.pdf]

S2 Table. Association of CHD and IS PRS with incident ASCVD, CHD, and IS in the first 10 years of follow-up, after adjusting for traditional risk factors, ABI, cIMT, and carotid plaque.

	Model 1: ASCVD outcomes, age + gender + TRFs + ABI + cIMT + carotid plaque + CHD PRS + IS PRS	
	**HR (95% CI)**	**P-value**
European Americans (Ncases=273, N=5398)		
CHD_PRS	1.53 (1.36, 1.73)	**1.35E-12**
IS_PRS	1.37 (1.21, 1.54)	**3.90E-07**
	Model 2: CHD outcomes, age + gender + TRFs + ABI + cIMT + carotid plaque + CHD PRS + IS PRS	
	**HR (95% CI)**	**P-value**
European Americans (Ncases=220, N=5398)		
CHD_PRS	1.57 (1.37, 1.79)	**2.37E-11**
IS_PRS	1.08 (0.94, 1.24)	0.268
	Model 3: IS outcomes, age + gender + TRFs + ABI + cIMT + carotid plaque + CHD PRS +IS PRS	
	**HR (95% CI)**	**P-value**
European Americans (Ncases=64, N=5398)		
CHD_PRS	1.43 (1.12, 1.82)	**0.00426**
IS_PRS	3.17 (2.48, 4.05)	**< 2.20E-16**
