# Supplementary material for: Coronary heart disease and ischemic stroke polygenic risk scores and atherosclerotic cardiovascular disease in a diverse, population-based cohort study
Source: PLoS One. 2023 Jun 16;18(6):e0285259. doi: 10.1371/journal.pone.0285259 (PMC10275447; doi:10.1371/journal.pone.0285259)
Supplement: S3 Table — Reclassification, given by the net reclassification index (NRI) and integrated discrimination index (IDI), and changes in discrimination upon the addition of the CHD PRS, IS PRS, ABI, cIMT, and carotid plaque to models with traditional risk factors in White participants. (PDF) [file pone.0285259.s009.pdf]

S3 Table. Association of ABI, cIMT, and carotid plaque with incident ASCVD in the first 10 years of follow-up, adjusting for traditional risk factors and the CHD and IS PRS. Reclassification, given by the net reclassification index (NRI) and integrated discrimination index (IDI), and changes in discrimination upon the addition of the CHD PRS, IS PRS, ABI, cIMT, and carotid plaque to models with traditional risk factors in White participants.

A: Association of ABI, cIMT, and carotid plaque with incident ASCVD in the first 10 years of follow-up, adjusting for traditional risk factors.

	Model 1: ASCVD outcomes, age + gender + TRFs	
	**HR (95% CI)**	**P-value**
European Americans (Ncases=273, N=5398)		
ABI	0.82 (0.35, 1.96)	0.659
cIMT	2.54 (1.15, 5.61)	**0.021**
Carotid plaque	1.87 (1.45, 2.41)	**1.18E-06**

B: Association of ABI, cIMT, and carotid plaque with incident ASCVD in the first 10 years of follow-up, adjusting for traditional risk factors and the CHD and IS PRS.

	Model 2: ASCVD outcomes, age + gender + TRFs + CHD PRS + IS PRS	
	**HR (95% CI)**	**P-value**
European Americans (Ncases=273, N=5398)		
ABI	1.00 (0.42, 2.36)	0.999
cIMT	2.70 (1.17, 6.24)	**0.020**
Carotid plaque	1.75 (1.35, 2.25)	**1.70E-05**

C: Reclassification, given by the NRI and IDI, and changes in discrimination upon the addition of the CHD PRS, IS PRS, ABI, cIMT, and carotid plaque to models with traditional risk factors in White and Black Participants.

**White participants**				
	**Time to first ASCVD event ~ established risk factors + ABI + cIMT + carotid plaque**			
**NRI**	**Estimate**	**95% CI**	**IDI (95% CI)**	**c-statistic difference**
NRI	0.100	(0.008, 0.183)	0.013 (0.008, 0.026)	0.016
NRI +	0.077	(-0.006, 0.150)		
NRI -	0.023	(0.005, 0.042)		
	**Time to first ASCVD event ~ established risk factors + CHD PRS**			
**NRI**	**Estimate**	**95% CI**	**IDI (95% CI)**	**c-statistic difference**
NRI	0.114	(0.026, 0.191)	0.020 (0.010, 0.034)	0.018
NRI +	0.095	(0.016, 0.169)		
NRI -	0.018	(0.003, 0.036)		
	**Time to first ASCVD event ~ established risk factors + IS PRS**			
**NRI**	**Estimate**	**95% CI**	**IDI (95% CI)**	**c-statistic difference**
NRI	0.044	(-0.035, 0.120)	0.011 (0.003, 0.024)	0.007
NRI +	0.030	(-0.041, 0.105)		
NRI -	0.014	(-0.005, 0.025)		
	**Time to first ASCVD event ~ established risk factors + ABI + cIMT + carotid plaque + CHD PRS**			
**NRI**	**Estimate**	**95% CI**	**IDI (95% CI)**	**c-statistic difference**
NRI	0.176	(0.085, 0.272)	0.031 (0.021, 0.051)	0.034
NRI +	0.145	(0.064, 0.236)		
NRI -	0.030	(0.012, 0.052)		
	**Time to first ASCVD event ~ established risk factors + ABI + cIMT + carotid plaque + IS PRS**			
**NRI**	**Estimate**	**95% CI**	**IDI (95% CI)**	**c-statistic difference**
NRI	0.117	(0.036, 0.216)	0.024 (0.014, 0.044)	0.022
NRI +	0.088	(0.011, 0.184)		

NRI -	0.028	(0.009, 0.045)

These analyses were restricted to a subset of participants with subclinical atherosclerosis data available.
